# A conserved role of the insulin-like signaling pathway in diet-dependent uric acid pathologies in *Drosophila melanogaster*

**DOI:** 10.1371/journal.pgen.1008318

**Published:** 2019-08-15

**Authors:** Sven Lang, Tyler A. Hilsabeck, Kenneth A. Wilson, Amit Sharma, Neelanjan Bose, Deanna J. Brackman, Jennifer N. Beck, Ling Chen, Mark A. Watson, David W. Killilea, Sunita Ho, Arnold Kahn, Kathleen Giacomini, Marshall L. Stoller, Thomas Chi, Pankaj Kapahi

**Affiliations:** 1 The Buck Institute for Research on Aging, Novato, California, United States of America; 2 Davis School of Gerontology, University of Southern California, Los Angeles, California, United States of America; 3 Department of Bioengineering and Therapeutic Sciences, University of California San Francisco, San Francisco, California, United States of America; 4 Division of Biomaterials and Bioengineering, University of California San Francisco, San Francisco, California, United States of America; 5 Nutrition & Metabolism Center, Children’s Hospital Oakland Research Institute, Oakland, California, United States of America; 6 Department of Urology, University of California San Francisco, San Francisco, California, United States of America; The Scripps Research Institute, UNITED STATES

## Abstract

Elevated uric acid (UA) is a key risk factor for many disorders, including metabolic syndrome, gout and kidney stones. Despite frequent occurrence of these disorders, the genetic pathways influencing UA metabolism and the association with disease remain poorly understood. In humans, elevated UA levels resulted from the loss of the of the urate oxidase (*Uro*) gene around 15 million years ago. Therefore, we established a *Drosophila melanogaster* model with reduced expression of the orthologous *Uro* gene to study the pathogenesis arising from elevated UA. Reduced *Uro* expression in *Drosophila* resulted in elevated UA levels, accumulation of concretions in the excretory system, and shortening of lifespan when reared on diets containing high levels of yeast extract. Furthermore, high levels of dietary purines, but not protein or sugar, were sufficient to produce the same effects of shortened lifespan and concretion formation in the *Drosophila* model. The insulin-like signaling (ILS) pathway has been shown to respond to changes in nutrient status in several species. We observed that genetic suppression of ILS genes reduced both UA levels and concretion load in flies fed high levels of yeast extract. Further support for the role of the ILS pathway in modulating UA metabolism stems from a human candidate gene study identifying SNPs in the ILS genes *AKT2* and *FOXO3* being associated with serum UA levels or gout. Additionally, inhibition of the NADPH oxidase (*NOX*) gene rescued the reduced lifespan and concretion phenotypes in *Uro* knockdown flies. Thus, components of the ILS pathway and the downstream protein NOX represent potential therapeutic targets for treating UA associated pathologies, including gout and kidney stones, as well as extending human healthspan.

## Author summary

Enzymatic purine degradation in humans ends with uric acid (UA). Multiple genetic and dietary factors raise UA levels above the norm, which is called hyperuricemia or hyperuricosuria when detected in the serum or urine, respectively. Clinical studies report a correlation between elevated UA and a plethora of chronic diseases including crystalopathies like UA kidney stones and gout, or metabolic and vascular disorders such as diabetes, obesity, and coronary artery disease. Here, we identified a regulatory role for the insulin-like signaling cascade affecting UA metabolism using a *Drosophila melanogaster* model. In the process we determined previously unrecognized potential drug targets to treat elevated UA levels and associated pathologies such as gout or UA kidney stones, with the potential additional benefit of extending human healthspan. Our work also establishes the fly as a model system to characterize the influence of genetic and dietary factors in gout or UA kidney stone development in a manner readily amenable for small-scale screening of drug interventions. The novelty of our findings, their broad impact, and relevance for multiple diseases opens up an important area of research to define mechanisms of UA accumulation.

## Introduction

Purine homeostasis represents a conserved metabolic pathway that is sustained by multiple enzymes orchestrating *de novo* synthesis, salvage, and degradation of purine intermediates. Urate oxidase, which is conserved across species, catalyzes one of the last steps of purine degradation converting uric acid (UA) to allantoin. However, due to multiple point mutations in the urate oxidase gene (*Uro*) human ancestors lost the ability to synthesize a functional urate oxidase, thus, increasing serum and urinary UA levels [1–5]. While most mammals show serum UA levels of 1 mg/dl and lower, healthy humans generally are in the range of 4–6 mg/dl, close to the UA solubility limit of 6.8 mg/dl at physiological pH and body temperature [6, 7]. Despite a regulatory network balancing UA production and excretion, UA levels increase due to both age and a nutrient-rich diet [8]. Several genetic risk factors are associated with increased UA levels including genes of purine homeostasis, glucose metabolism, or UA transporters. Dietary risk factors are sugar-sweetened beverages, alcohol, red meat, and seafood, all found in over-abundance in the Western diet [8, 9].

In humans, elevated UA levels in the blood (hyperuricemia) or urine (hyperuricosuria) are key risk factors for crystalopathies such as gout and kidney stones, metabolic syndrome, as well as premature death and a higher all-cause mortality risk [10–16]. Alarmingly, the prevalence of hyperuricemia in the US population is over 20% [17, 18]. Different treatment options are commonly used to lower UA levels. As first-line therapy, an inhibitor of xanthine dehydrogenase such as allopurinol is prescribed to prevent the xanthine dehydrogenase mediated oxidation of xanthine to UA. Further options include the use of uricosuric drugs such as probenecid to increase renal UA excretion or administration of a recombinant urate oxidase enzyme called pegloticase degrading extracellular UA to the water-soluble allantoin [19, 20]. Common to the UA lowering therapies are the well-documented issues of adverse drug reactions, contraindications, relevant drug-drug interactions, and the need for anti-inflammatory prophylaxis [9]. Interestingly, cohort studies also link exceptional longevity and a reduced prevalence of age-related diseases with UA levels at the lower end of the human serum UA spectrum [21, 22]. In sum, the data correlate elevated UA levels with a higher prevalence of crystalopathies as well as a shortened healthspan and lifespan. However, the molecular mechanisms by which UA mediates negative health outcomes are still a matter of debate.

*Drosophila melanogaster* provides an excellent model system to study both crystalopathies and lifespan [23–27]. Crystalopathies in flies can be induced either by feeding lithogenic agents such as ethylene glycol [28–32] or via genetic inactivation of xanthine dehydrogenase [33]. The resulting crystals usually accumulate in the Malpighian tubules, the invertebrate homolog of the human kidney convoluted tubules, and therefore recapitulate the clinical pictures of calcium oxalate urolithiasis and xanthinuria, respectively [34]. In addition, aging studies employing *Drosophila* take advantage of its short lifespan and fast development. Many reports highlight the relevance of nutrient-sensing pathways such as the insulin-like signaling (ILS) cascade in extending the organism lifespan [35–37]. Yet, there is currently no *Drosophila* model addressing the impact of elevated UA levels on crystalopathies and lifespan. Therefore, we designed a fly model to study the effects of elevated UA levels by recapitulating both aspects encountered in hominoid evolution: depletion of urate oxidase activity combined with changing dietary habits. Our model showed a diet-dependent accumulation of UA and shortening of lifespan. Using this model, we identified the evolutionarily conserved ILS pathway and the downstream factors FoxO and NADPH oxidase to reduce UA and its associated pathologies which might provide new targets for UA lowering therapies.

## Results and discussion

### *Uro* knockdown shortens lifespan in a diet-dependent manner in *Drosophila melanogaster*

**Fig 1:**
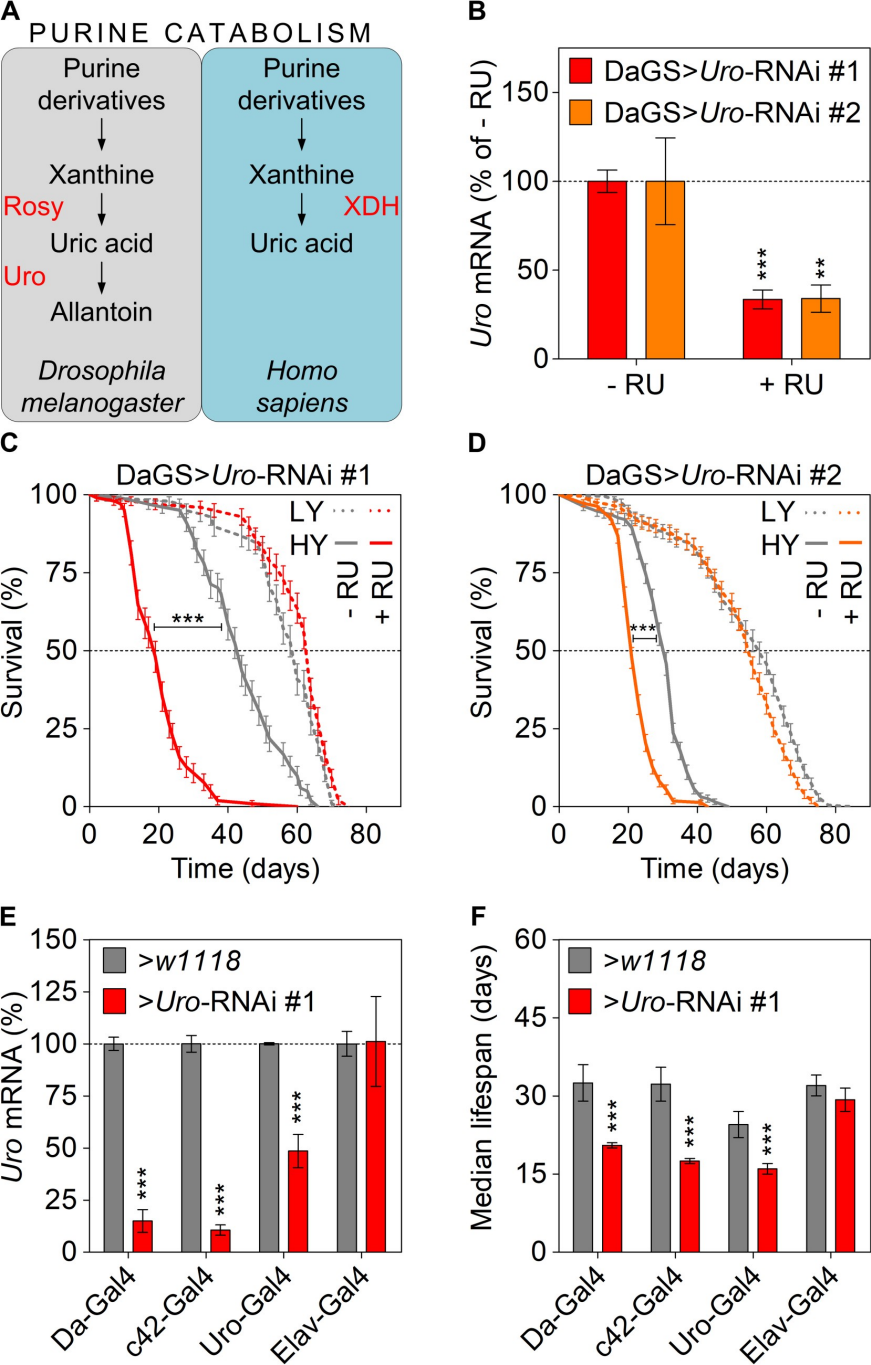
*Uro* knockdown attenuates lifespan on a high yeast diet. (A) Schematic comparison of the purine catabolism of *Drosophila melanogaster* and humans with relevant enzymes in red and metabolites in black. (B) *Uro* mRNA levels were determined in presence and absence of RU486 by qRT-PCR. Progeny of crosses combining the *daughterless* gene switch (DaGS) driver with one of the two different *Uro* targeting RNAi constructs (*Uro*-RNAi #1, *Uro*-RNAi #2) were fed diets without RU486 (- RU) or with RU486 (+ RU). For each cross *Uro* mRNA value of the - RU diet was set as 100%. (C, D) Kaplan-Meier survival curves of DaGS>*Uro*-RNAi #1 (C) and DaGS>*Uro*-RNAi #2 (D) flies fed diets containing a low (LY; 0.5%) or high yeast extract content (HY; 5%) +/- RU. (E) Relative *Uro* mRNA levels of different Gal4 driver lines (*Da-GAL4*, *c42-GAL4*, *Uro-GAL4*, *Elav-GAL4*) crossed with the background control *w1118* or the *Uro*-RNAi #1. For each cross with *w1118* the *Uro* mRNA value was set to 100%. (F) Average median lifespan of flies from (E) fed the HY diet. The average median lifespan was deduced from multiple survival curves and is defined as the time point in days when 50% of the population is alive. Error bars represent the standard error (SE). With the exception of the LY conditions in (D) all experiments were run in independent biological repeats. Supporting information is given in S1 Fig.

Unlike humans, *Drosophila melanogaster* uses the enzyme urate oxidase to convert UA into allantoin (Fig 1A). Therefore, we “humanized” *Drosophila* to recapitulate the lack of functional urate oxidase making UA the end-product of purine catabolism. We used the heterologous, ligand (RU486)-induced gene switch system to generate spatially and temporally precise RNAi-mediated knockdown of *Uro* in *Drosophila* [38]. Thus, flies carrying the ubiquitous *daughterless* gene switch *GAL4* driver (DaGS) were crossed to flies with one of the two different UAS-*Uro*-RNAi responder transgenes (*Uro*-RNAi #1 or #2). 2–3 day-old progeny (DaGS>*Uro*-RNAi) of such crosses were then fed diets supplemented with the Gal4 activating ligand RU486 (+ RU) to induce the *Uro* knockdown or the corresponding vehicle control (- RU). Compared to the isogenic control siblings, flies consuming the + RU diet showed for both RNAi constructs a 70% knockdown of the *Uro* mRNA (Fig 1B). To mimic the change in dietary habits of Western civilization flies were fed diets with different concentrations of yeast extract. In comparison to their isogenic control siblings, i.e. DaGS>*Uro*-RNAi #1 flies reared on a diet without RU486, the knockdown of *Uro*, i.e. DaGS>*Uro*-RNAi #1 flies fed a diet supplemented with RU486, did not affect the median or total lifespan of flies consuming a low yeast (LY) diet containing 0.5% yeast extract (Fig 1C and 1D, S1 Fig). However, when *Uro* knockdown flies were fed a high yeast (HY) diet containing 5% yeast extract their lifespan was significantly shorter compared to the control siblings receiving the same food, but without RU486 supplementation (Fig 1C and 1D). We also compared the median lifespan of DaGS>*Uro*-RNAi #1 flies on different yeast concentrations, showing that the impact of urate oxidase depletion on survival becomes apparent at a dietary yeast concentration of 3% and above (S1 Fig). At 3% yeast concentration the median lifespan of control flies (DaGS>*Uro*-RNAi #1 flies reared without RU486) was 48 days, whereas the median lifespan of their *Uro* knockdown siblings (DaGS>*Uro*-RNAi #1 flies reared with RU486) was 31.5 days, a reduction of 34%. Similarly, at 4% yeast concentration the median lifespans of control and knockdown flies were 45 and 27 days, respectively, a decrease of 40%. At 5% yeast concentration the difference in median lifespan was even more pronounced comparing 39.4 days for control flies and 20.5 days for the *Uro* knockdown flies, which represented a shortening of 48%. The latter value represented the plateau, given that a higher dietary yeast concentration of 8% did not further augment the difference in median lifespan between control (38 days) and *Uro* knockdown (20 days) flies (S1 Fig). In other words, we observed an inverse correlation between the dietary yeast content and the median lifespan with the *Uro* knockdown flies being more susceptible to higher yeast concentrations than controls.

To confirm our findings and demonstrate tissue-specificity as well as independence of the lifespan effect from the RU486 ligand, we used the ubiquitous (*Da-GAL4*) or Malpighian tubule-specific (*c42-GAL4*, *Uro-GAL4*) driver lines not relying on RU486 for activation. Considering that Malpighian tubules are the main tissue expressing *Uro* the neuronal *Elav-GAL4* driver was used as a negative control [39]. For the four non-gene switch driver lines crosses to the *Uro*-RNAi and *w1118*, the genetic background flies used to outcross all driver and other transgenic lines, were performed in parallel. As expected, compared to the individual *w1118* control crosses efficient *Uro* knockdown was achieved only when using the *Da-GAL4*, *c42-GAL4* and *Uro-GAL4* drivers (Fig 1E). Coinciding with the *Uro* knockdown, the shortened lifespan of flies consuming the HY diet was only observed with the driver lines actively reducing *Uro* expression but was absent in case of the *Elav-GAL4* driver (Fig 1F). Importantly, flies with reduced *Uro* expression were significantly longer lived on low nutrient diets with 0.5% and 1% yeast extract compared to diets with a higher yeast extract content (Fig 1C and 1D, S1 Fig), demonstrating the importance of diet in UA mediated effects on lifespan.

### *Uro* knockdown enhances UA accumulation in a diet-dependent manner in *Drosophila*

**Fig 2:**
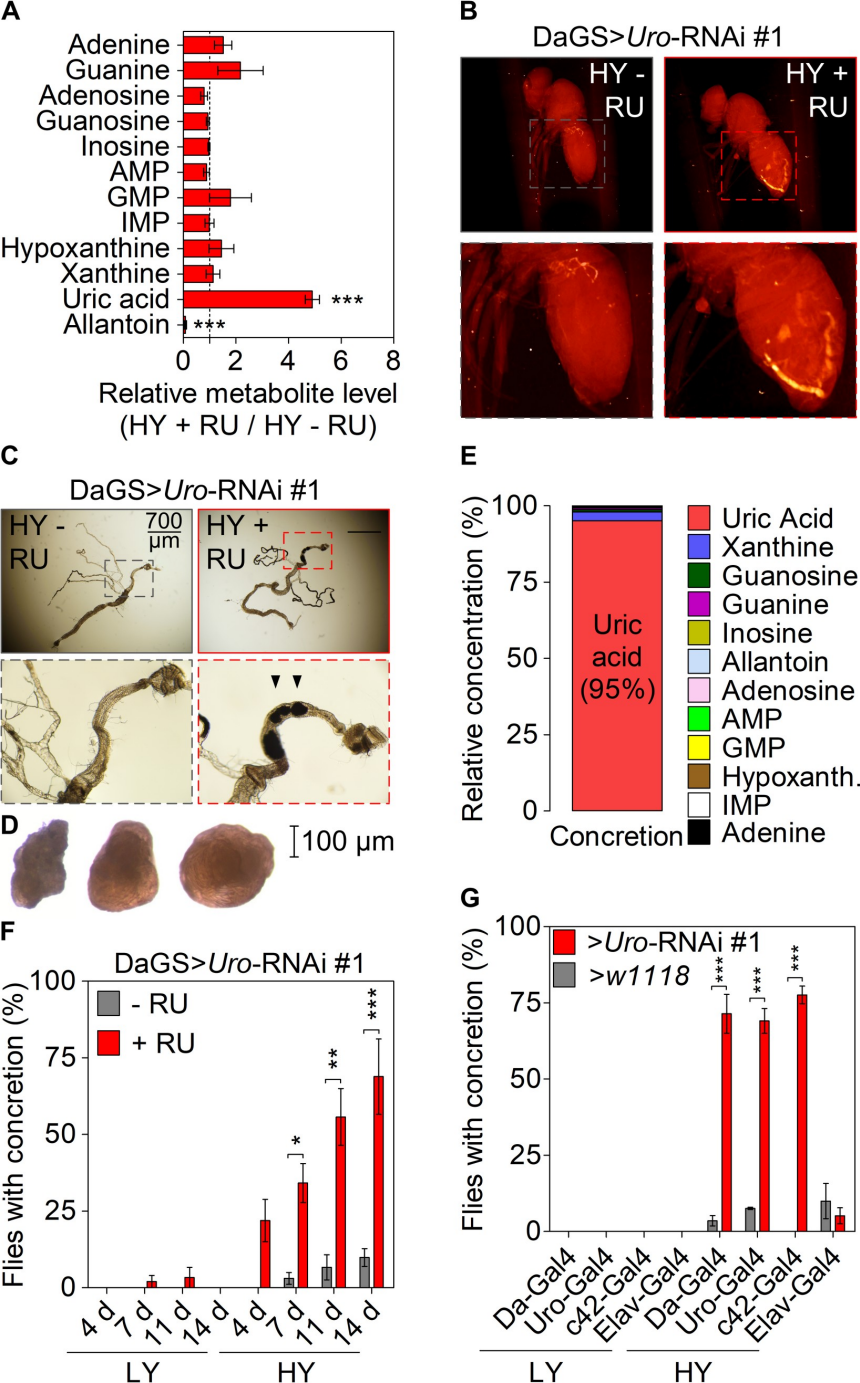
*Uro* knockdown enhances UA concretion formation on a high yeast diet. (A) Mass spectrometric analysis of purine metabolite concentrations of DaGS>*Uro*-RNAi #1 fly homogenates comparing siblings fed the HY + RU and HY - RU diet for 14 days. (B) Micro-CT images of intact flies from (A). Fly length is ~2 mm. (C) Guts with the attached Malpighian tubules were dissected from flies in (B). Black arrowheads point to solid aggregates (concretions) in the hindgut region. (D) Light microscopic pictures of concretions extracted from the hindgut lumen of DaGS>*Uro*-RNAi #1 flies fed the HY diet + RU for 14 days. (E) Metabolomic analysis of the same purine metabolites as in (A) of extracted concretions from *Uro* knockdown flies. (F) The extent and kinetic of concretion formation in DaGS>*Uro*-RNAi #1 flies fed the LY or HY diet +/- RU for 4 to 14 days was determined by microscopic analysis after dissection of the hindgut. (G) Concretion formation of flies from Fig 1E fed the LY or HY diet for 14 days was determined by microscopic analysis after dissection of the hindgut. Error bars represent the SE of multiple biological repeats. Further supporting information is shown in S2 Fig.

Next, using *Uro* knockdown flies (DaGS>*Uro*-RNAi #1 + RU486) in comparison to their isogenic control siblings (DaGS>*Uro*-RNAi #1 - RU486) metabolomic profiling verified the elevation of UA levels after feeding the HY diets for 14 days. The fivefold increase in UA levels in the *Uro* knockdown flies was accompanied by an almost complete loss of allantoin production (Fig 2A). In accordance with the qRT-PCR (Fig 1B), these data indicate on the metabolite level the efficiency of urate oxidase depletion (Fig 2A). An unbiased micro-CT scan revealed radiopaque masses (putative ‘concretions') specifically in the abdominal area of *Uro* knockdown flies, but not in the control population (Fig 2B). This observation was further validated by optical microscopy of dissected animals revealing the presence of concretions in the Malpighian tubule and hindgut of *Uro* knockdown flies (Fig 2C). Due to the restricted expression pattern of the *Uro* gene to the excretory system both the Malpighian tubule and the hindgut represent the anatomical sites where UA is most likely expected to accumulate [40]. Furthermore, metabolomic analyses of isolated concretions (Fig 2D) confirmed UA as the predominant component, accounting for 95% of the metabolites measured (Fig 2E). We found enhanced UA aggregation with increasing age (Fig 2F). While DaGS>*Uro*-RNAi flies without RU486 supplementation formed almost no concretions, in *Uro* knockdown flies their appearance was observed as early as 4 days of feeding the HY diet. The proportion of flies forming UA deposits increased gradually over time with almost 70% of *Uro* knockdown flies forming concretions after 14 days (Fig 2F). In contrast, DaGS>*Uro*-RNAi #1 flies fed the LY diet, with or without RU486, hardly formed any concretion (Fig 2F). Very much like the lifespan phenotype, we investigated the concretion formation with the set of non-gene switch drivers used before. Again, in comparison to the *w1118* control crosses performed in parallel a high rate of concretion formation was only detectable on a HY diet when the *Uro*-RNAi construct was expressed by the *Da-GAL4*, *c42-GAL4* or *Uro-GAL4* driver, thus causing efficient urate oxidase depletion and UA accumulation (Fig 2G). The *w1118* or *Uro*-RNAi crosses with the *Elav-GAL4* driver did not result in concretion formation underscoring the tissue-specificity of the effect. Using the high proportion (~ 70%) of *Uro* knockdown flies developing concretions after feeding the HY diet for 14 days as a reference, we analyzed the dietary yeast concentration as factor in promoting concretion formation. Like the lifespan attenuation, concretion formation started to increase significantly in *Uro* knockdown flies when the dietary yeast concentration reached 3% or more (S2A Fig). Concretion formation was also seen when comparing the control siblings and knockdown flies of the DaGS>*Uro*-RNAi #2 population (S2B Fig). However, DaGS>*Uro*-RNAi #2 flies reared on the HY diet without RU486 supplementation showed a much higher background in terms of concretion formation (cf. S2A and S2B Fig). In analogy to human pathophysiology the shortened lifespan and enhanced concretion formation of *Uro* knockdown flies match with the enhanced mortality in patients with gout as well as the age- and diet-dependent occurrence of crystalopathies in humans [16, 41–43].

### The dietary purine content mediates the lifespan and concretion phenotypes of *Uro* knockdown flies

**Fig 3:**
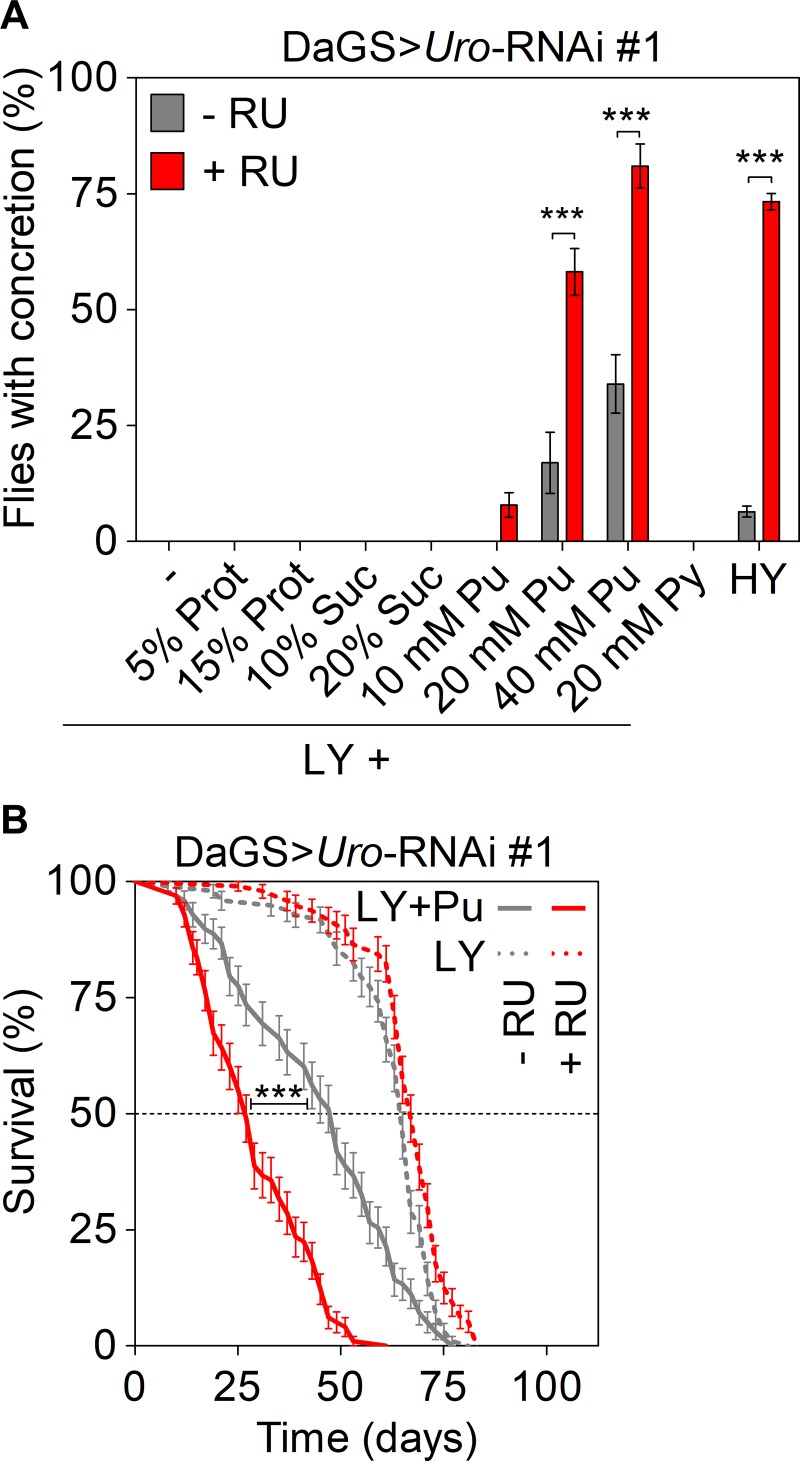
Dietary purine mediates lifespan attenuation and UA concretion formation. (A) Concretion formation of DaGS>*Uro*-RNAi #1 flies reared on a HY or LY diet for 14 days. Where indicated, the latter was supplemented with increasing concentrations of protein (Prot), sucrose (Suc), purine (Pu) or pyrimidine (Py). Each food was generated in a - RU and + RU version to compare concretion formation of isogenic control and *Uro* knockdown siblings, respectively. (B) Survival curve of DaGS>*Uro*-RNAi #1 flies fed the LY diet without or with 40 mM purine supplementation (+ Pu) in presence or absence of RU.

To determine what component of the HY diet triggered increased UA production and associated phenotypes, we systematically supplemented the ‘benign’ LY diet with various basic nutrient groups: purines, pyrimidines, proteins, or sugar. Of the components tested, we found that purines (metabolic precursors of UA), but not pyrimidines, proteins, or sugar led to a dose-dependent increase in concretion formation when added to the LY food (Fig 3A). Dietary purine supplementation above 10 mM caused a significantly higher concretion formation in *Uro* knockdown flies (DaGS>*Uro*-RNAi #1 with RU486) compared to their isogenic controls (DaGS>*Uro*-RNAi #1 without RU486). Interestingly, purine supplementation of 20 and 40 mM even caused concretion formation to rise in the DaGS>*Uro*-RNAi #1 flies without RU486 (Fig 3A), thereby showing the harmful and lithogenic potential of dietary purines. Given that the purine supplemented LY diet phenocopied the concretion formation seen with the HY diet, we examined the impact of purines on lifespan. Intriguingly, addition of 40 mM purines to the LY diet (LY+Pu) recapitulated the lifespan effects seen with the HY diet (cf. Figs 3B and 1C). I.e., purine addition caused a) a shortening of the lifespan of control flies and b) this lifespan attenuation was more pronounced in *Uro* knockdown flies, which are unable to convert the increased UA load to allantoin (Fig 3B). Thus, altering the ability to utilize purines could be an effective way to prevent UA associated pathologies in flies and strengthen the relevance of the *Uro* knockdown model as translational research tool.

### Manipulation of purine homeostasis rescues the phenotypes of *Uro* knockdown flies

**Fig 4:**
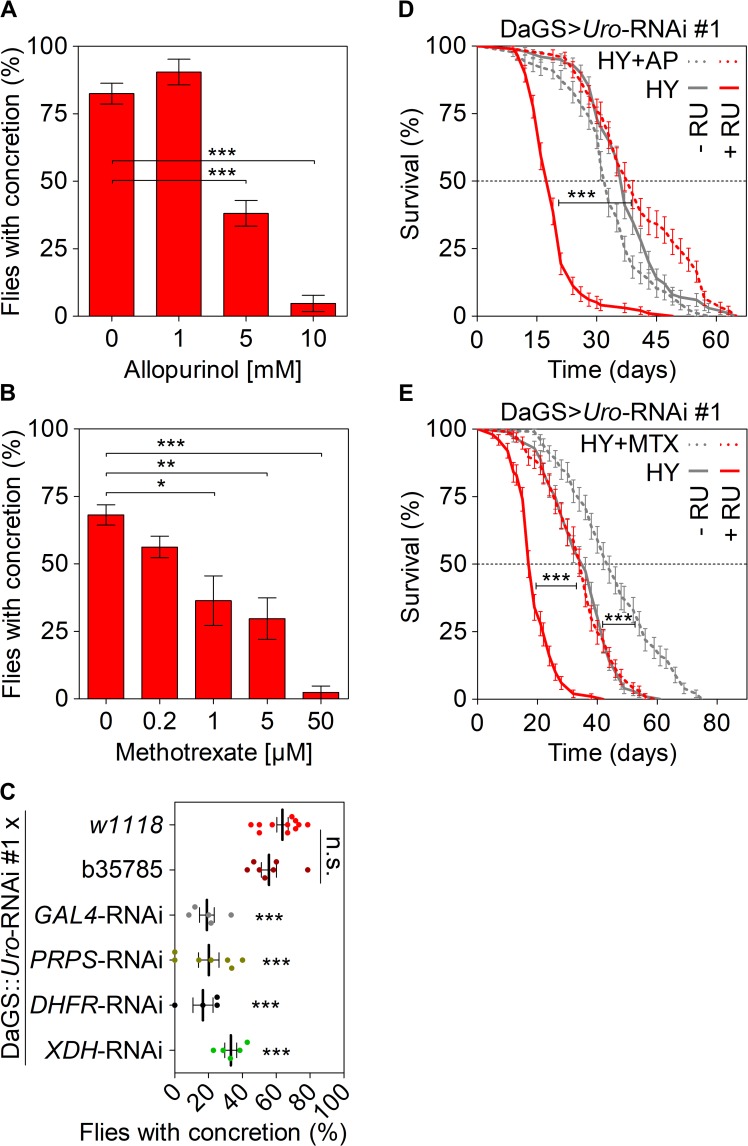
Modulation of purine homeostasis rescues the lifespan attenuation and UA concretion formation of *Uro* knockdown flies. (A, B) Concretion formation of DaGS>*Uro*-RNAi #1 flies after 14 days of feeding a HY + RU diet supplemented with the indicated concentration of allopurinol (A) or methotrexate (B). (C) The recombinant DaGS::*Uro*-RNAi #1 line was crossed to *w1118* (no additional UAS-locus), b35785 (carrying a no target UAS-mCherry-RNAi), or active UAS-RNAi lines targeting the transcription factor *GAL4*, *PRPP synthetase* (*PRPS*), *dihydrofolate reductase* (*DHFR*), or *xanthine dehydrogenase* (*XDH*). To measure concretion formation the flies were fed the HY + RU diet for 14 days prior to dissection. (D) Survival curve of DaGS>*Uro*-RNAi #1 flies fed the HY diet without or with 5 mM AP (+AP) supplementation in presence or absence of RU. (E) As in (D), but 5 **μ**M MTX (+MTX) supplementation. Error bars represent the SE of multiple biological repeats. Supporting data is shown in S3 Fig.

Purine homeostasis is controlled through orchestrated steps involving *de novo* synthesis, salvage, and degradation of purine intermediates (S3A Fig). Two drugs, allopurinol and methotrexate, are well-known to effectively perturb purine metabolism. Allopurinol is commonly used in the treatment of gout in humans and prevents purine degradation by inhibiting xanthine dehydrogenase (XDH), an enzyme that converts xanthine and hypoxanthine to UA. Methotrexate interferes with *de novo* purine synthesis by inhibiting dihydrofolate reductase (DHFR), an enzyme required for folate production (S3A Fig). Adding increasing amounts of allopurinol (Fig 4A) or methotrexate (Fig 4B) to the HY diet consumed by *Uro* knockdown flies caused a dose-dependent reduction of the concretion formation. At concentrations of 10 mM allopurinol or 50 μM methotrexate concretion formation was almost completely suppressed. We also determined if the effects of drug supplementation on food palatability and its consumption confounded our results. We compared food consumption of flies by a dye-colored food intake assay after them being fed a certain diet for 14 days. We did not observe a statistically significant change in food intake of *Uro* knockdown flies in case the HY diet was supplemented with allopurinol or methotrexate, when analyzed by ANOVA and Tukey’s multiple comparison post-test (S3B Fig). Next, we used a genetic strategy to verify known and identify novel targets that influence UA pathologies. Therefore, we generated a recombinant fly strain that harbors the DaGS driver and the *Uro*-RNAi #1 transgene, hereafter denoted by DaGS::*Uro*-RNAi. We confirmed that the recombinant DaGS::*Uro*-RNAi line when crossed to either *w1118* or b35785 (a strain that carries a no target UAS-mCherry-RNAi construct) still forms concretions when fed the RU486 supplemented HY diet. Indeed, 60–65% of the progeny of both crosses (DaGS::*Uro*-RNAi x *w1118* and DaGS::*Uro*-RNAi x b35785) form concretions after being fed the RU486 supplemented HY diet for 14 days (Fig 4C). Using a *GAL4*-RNAi line targeting expression of the *GAL4* transcription factor itself as a positive control reduced concretion formation of DaGS::*Uro*-RNAi x *GAL4*-RNAi flies due to the suppression of urate oxidase depletion (Fig 4C). Hence, the recombinant DaGS::*Uro*-RNAi line represents a useful tool to study UA associated pathologies.

In agreement with the allopurinol and methotrexate treatments, knocking down the corresponding target genes, *XDH* and *DHFR*, by RNAi also reduced concretion formation in the recombinant DaGS::*Uro*-RNAi flies (Fig 4C). Those data were complemented by knockdown of another crucial gene involved in purine *de novo* synthesis, 5'-phosphoribosyl-1'-pyrophosphate synthetase (*PRPS)*, whose knockdown by *PRPS*-RNAi reduced concretion formation of the DaGS::*Uro*-RNAi flies as efficiently as the *DHFR*-RNAi (Fig 4C, S3A Fig). Additionally, allopurinol and methotrexate were able to revert the short lifespan of DaGS>*Uro*-RNAi #1 flies when added to the RU486 supplemented HY diet (Fig 4D and 4E). Unlike allopurinol, methotrexate supplementation also triggered a lifespan extension of the control DaGS>*Uro*-RNAi #1 flies consuming the HY diet without RU486 (Fig 4E). Thus, the beneficial effect of methotrexate on lifespan is likely independent of the *Uro* expression level, yet, accentuates the importance of the purine metabolism in aging (Fig 4E). Overall, over-expression of purine homeostasis rescued the phenotypes of *Uro* knockdown flies, thereby supporting the validity of the fly model for UA based pathologies and suggesting an important role for purine metabolism in UA homeostasis.

### The transcription factor FoxO dampens concretion formation by reducing UA levels and ROS formation

**Fig 5:**
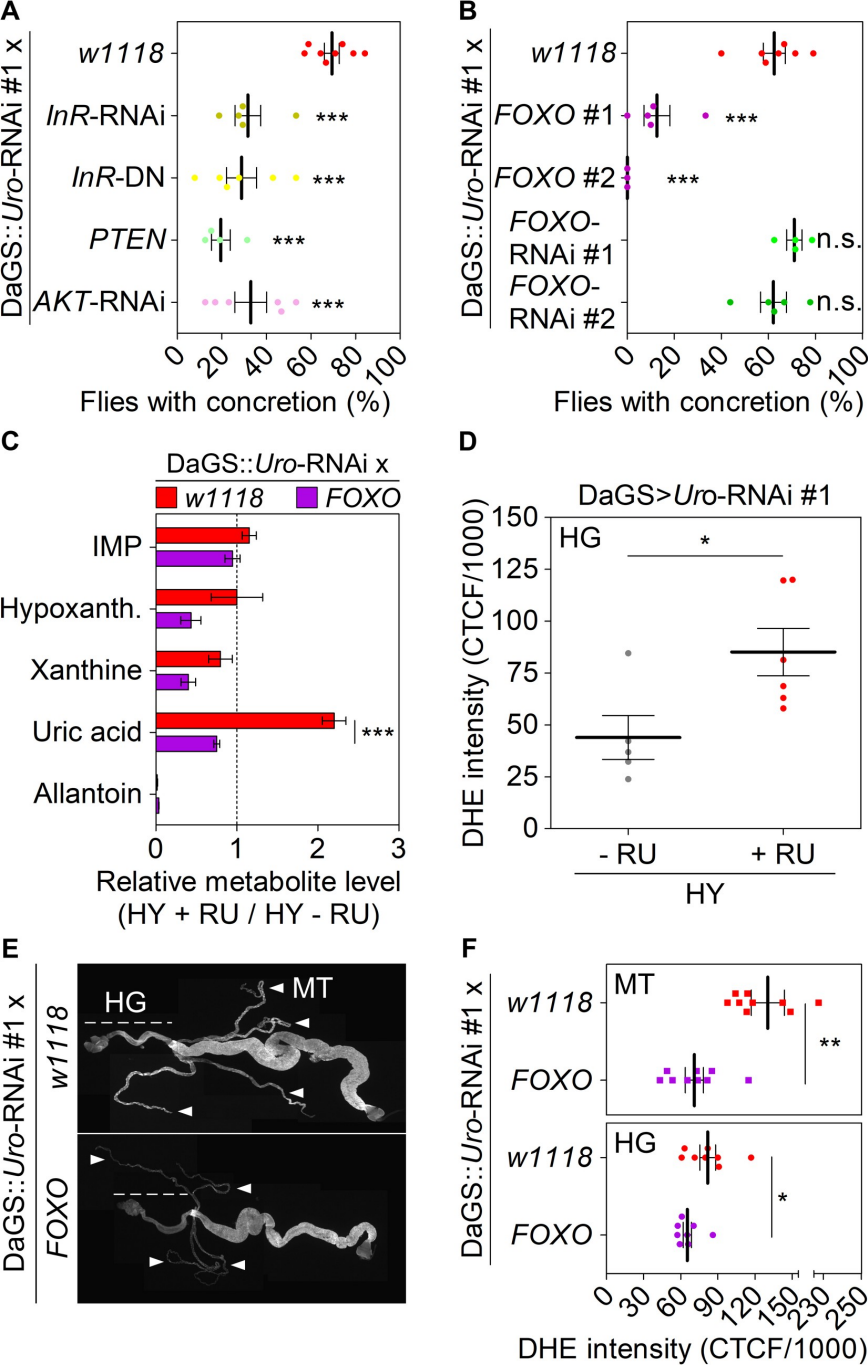
FoxO over-expression inhibits concretion formation by reducing UA levels and ROS formation. (A, B) The recombinant DaGS::*Uro*-RNAi #1 line was crossed to *w1118* (no additional UAS-locus), or strains with active UAS-transgenes triggering either inhibition of the insulin-like receptor (*InR*-RNAi, *InR*-DN (dominant negative)), AKT kinase (*AKT*-RNAi), or the transcription factor FoxO (*FOXO*-RNAi #1, *FOXO*-RNAi #2), or triggering over-expression of *PTEN*, or *FOXO* (*FOXO* #1, *FOXO* #2). Concretion formation was checked after 14 days of feeding the HY + RU diet. (C) Mass spectrometric analysis of purine metabolite concentrations of fly homogenates from DaGS::*Uro*-RNAi #1 crossed to *w1118* or *FOXO* #1. Metabolite levels were compared after 14 days of feeding flies the HY + RU or HY - RU diet. (D) ROS production of hindgut (HG) cells was determined by DHE staining. After 14 days on the HY + RU or HY - RU diet entire guts from DaGS>*Uro*-RNAi #1 flies were isolated and stained with DHE *ex vivo* before the corrected total cell fluorescence (CTCF) of hindgut cells was determined according to reference [72]. (E) Dissected guts and Malpighian tubules (MT) from flies in (C) reared on the HY + RU diet for 14 days were stained *ex vivo* with DHE and imaged to visualize reactive oxygen intermediates. Image stitching was used to combine multiple confocal images with overlapping fields of view to produce a panorama view in high-resolution. The HG and the four tubes of the MT are indicated by a dashed line and white arrowheads, respectively. (F) DHE staining intensity of the MT and HG was quantified as in (D). Error bars represent the SE of multiple biological repeats.

Common genetic variations identified to date explain only 6–7% of the variance encountered in serum UA levels [44–47]. Furthermore, commonly prescribed medications such as allopurinol can result in serious adverse drug reactions or provide insufficient efficacy in a high percentage of users [48–50]. Thus, other genes remain to be identified that may represent better targets to lower the metabolic UA load. To identify previously unrecognized regulators of UA metabolism, we started by examining the ILS pathway for two reasons. Firstly, ILS is a conserved signaling cascade activated when flies are fed a HY diet [25, 51–54]. Secondly, a recent genome-wide association study identified polymorphisms in the human ILS gene *IGF1R* that were associated with serum UA concentration [47]. Thus, we examined components of the ILS pathway as putative targets controlling UA production and utilization. We used the recombinant DaGS::*Uro*-RNAi line to address the impact of perturbing ILS signaling. Both strategies the RNAi mediated knockdown of the insulin-like receptor (*InR*) gene or expression of a dominant-negative *InR* gene variant (*InR*-DN) markedly reduced *Drosophila* concretion formation when fed the HY diet (Fig 5A). Similarly, over-expression of the phosphatase and tensin homolog (*PTEN*), which acts as negative regulator of ILS signaling downstream of the InR, significantly reduced concretion formation compared to the DaGS::*Uro*-RNAi x *w1118* flies (Fig 5A). Downstream of PTEN the kinase AKT integrates incoming ILS cues into metabolism by phosphorylating different effector molecules. Like inhibition of the InR, blocking ILS signaling by RNAi mediated depletion of AKT in *Uro* knockdown flies also reduced concretion formation (Fig 5A). One of the effector molecules inactivated by AKT phosphorylation is the evolutionarily conserved transcription factor FoxO [55]. Transgenic over-expression of *FOXO* in the background of the DaGS::*Uro*-RNAi flies showed a significant rescue of concretion formation with two different *FOXO* expression strains (Fig 5B). In contrast, knockdown of *FOXO* by two different RNAi constructs did not affect concretion formation (Fig 5B). In addition to over-expression and knockdown of *FOXO*, we examined the direct influence of UA levels on (endogenous) FoxO activity using the *Uro* knockdown flies. Based on the study by Alic *et al*., we picked seven target genes that are up- or down-regulated by binding of FoxO and are part of either purine metabolism (*Ade2*, *Ade5*, *Aprt*, *Veil*) or the ILS pathway (*AKT*, *InR*, *Thor*) [56]. No significant change in the expression level of the seven genes was observed when comparing control and *Uro* knockdown flies (S4A Fig). Thus, activity of FoxO was not altered in response to an increased UA level. However, as *FOXO* manipulation has a strong effect on UA concretion, we propose it is upstream of UA formation pathways. To further validate the physiological relevance of *FOXO* over-expression we compared the metabolite levels of relevant purine intermediates (including UA) in DaGS::*Uro*-RNAi x *w1118* and DaGS::*Uro*-RNAi x *FOXO* flies. While early intermediates of purine synthesis such as IMP were not altered, purine degradation products were reduced in flies with the *FOXO* over-expression (Fig 5C). In particular, UA levels were significantly reduced threefold, thus explaining the reduced deposit formation in flies over-expressing *FOXO*.

To determine whether the ILS pathway influences UA levels in a conserved manner between flies and humans, we assessed whether single nucleotide polymorphisms (SNPs) in different genes of the ILS pathway are associated with either serum UA (SUA) levels or gout in humans. Using data from 80,795 adult subjects from the Kaiser Permanente Research Program on Genes, Environment, and Health (S1 Table) [50], we found that SNPs in the *AKT2* and *FOXO3* genes were associated with either SUA levels or the incidence for gout (Table 1, S2 Table). This association was significant after adjusting for multiple testing of phenotypes and SNPs within each gene. In light of our fly studies identifying *AKT* inhibition (Fig 5A) and *FOXO* over-expression (Fig 5B) as suppressors of UA concretions the human SNP associations shown in Table 1 support the idea that modulation of the ILS signaling network could play a critical and eventually conserved role in affecting UA levels and associated pathology in both flies and humans.

**Table 1:** A human candidate-gene study (CGS) identifies SNPs in *FOXO3* and *AKT2* being associated with UA pathologies.

Human gene	Phenotype		Gene threshold
	Gout	SUA	
*FOXO1*	0.00611	0.04906	0.00058
***FOXO3***	0.01596	**0.00004**	0.00061
*AKT1*	0.07641	0.08287	0.00066
***AKT2***	**0.00003**	0.01738	0.00066
*AKT3*	0.00804	0.01533	0.00033
*PTEN*	0.01080	0.00979	0.00064

P-values of single nucleotide polymorphisms (SNPs) within relevant genes of the ILS network that are significantly associated with gout or serum uric acid (SUA) levels in human subjects are indicated in bold. Significance of calculated p-values is set by the gene-specific threshold (last column). Supporting information such as description of the study population and effect sizes for significant SNPs is given in S1 Table and S2 Table as well as the material and methods section.

Next, we addressed the underlying mechanism of the link between the ILS pathway and UA levels. UA has been proposed to play several physiological roles, depending on where it is acting. For instance, UA can act as an antioxidant in the blood, where it accounts for up to 60% of the antioxidative capacity [57]. In turn, in an intracellular setting such as in adipocytes, UA is considered a prooxidant and proinflammatory molecule [12, 58, 59]. Similarly, in *Drosophila* UA is considered a damage-associated molecular pattern (DAMP) able to trigger a so-called sterile inflammation, which typically results in the production of antimicrobial peptides [60]. Compared to their isogenic control siblings *Uro* knockdown flies depicted a two- to eightfold increase in the expression of the three antimicrobial peptides *attacin A* (*Att A*), *diptericin* (*Dipt*), and *metchnikowin* (*Metch*) after 14 days on the HY diet (S4B Fig). Increased expression of the antimicrobial peptides was seen for both crosses DaGS>*Uro*-RNAi #1 / #2 on the HY plus RU486 diet. Using the recombinant DaGS::*Uro*-RNAi line over-expression of *FOXO* diminished the high levels of antimicrobial peptides (S4C Fig). However, knockdown of the known NFκB paralogs *Relish*, *Dorsal* or the *Dorsal-related immunity factor* (*Dif*), whose gene products act as dimeric transcription factors activating expression of antimicrobial peptides, did not alleviate UA concretion formation (S4D Fig) [61, 62]. Work by Zhao *et al*. indicates that over-expression of individual antimicrobial peptides like diptericin increases the tolerance of flies resisting oxidative stress [63]. Thus, the high expression of antimicrobial peptides (S4B Fig) could represent a defense mechanism of *Uro* knockdown flies against oxidative stress rather than being a driver of UA accumulation. Consistent with this idea of intracellular UA generating a prooxidative milieu, *Uro* knockdown flies showed significantly elevated ROS levels in the cells of the hindgut as revealed by the doubling of the ROS sensitive DHE staining intensity (Fig 5D). Considering the well-documented role of FoxO proteins in cellular responses to combat oxidative stress [55, 64, 65], we speculated if *FOXO* over-expression could reduce the build-up of ROS found in *Uro* knockdown flies. Comparing ROS levels of DaGS::*Uro*-RNAi x *w1118* flies to DaGS::*Uro*-RNAi x *FOXO* flies showed that an increased FoxO abundance indeed caused a reduction of ROS in the hindgut as well as Malpighian tubules (Fig 5E and 5F).

### Lifespan shortening and UA concretion formation of *Uro* knockdown flies are mediated by NOX

**Fig 6:**
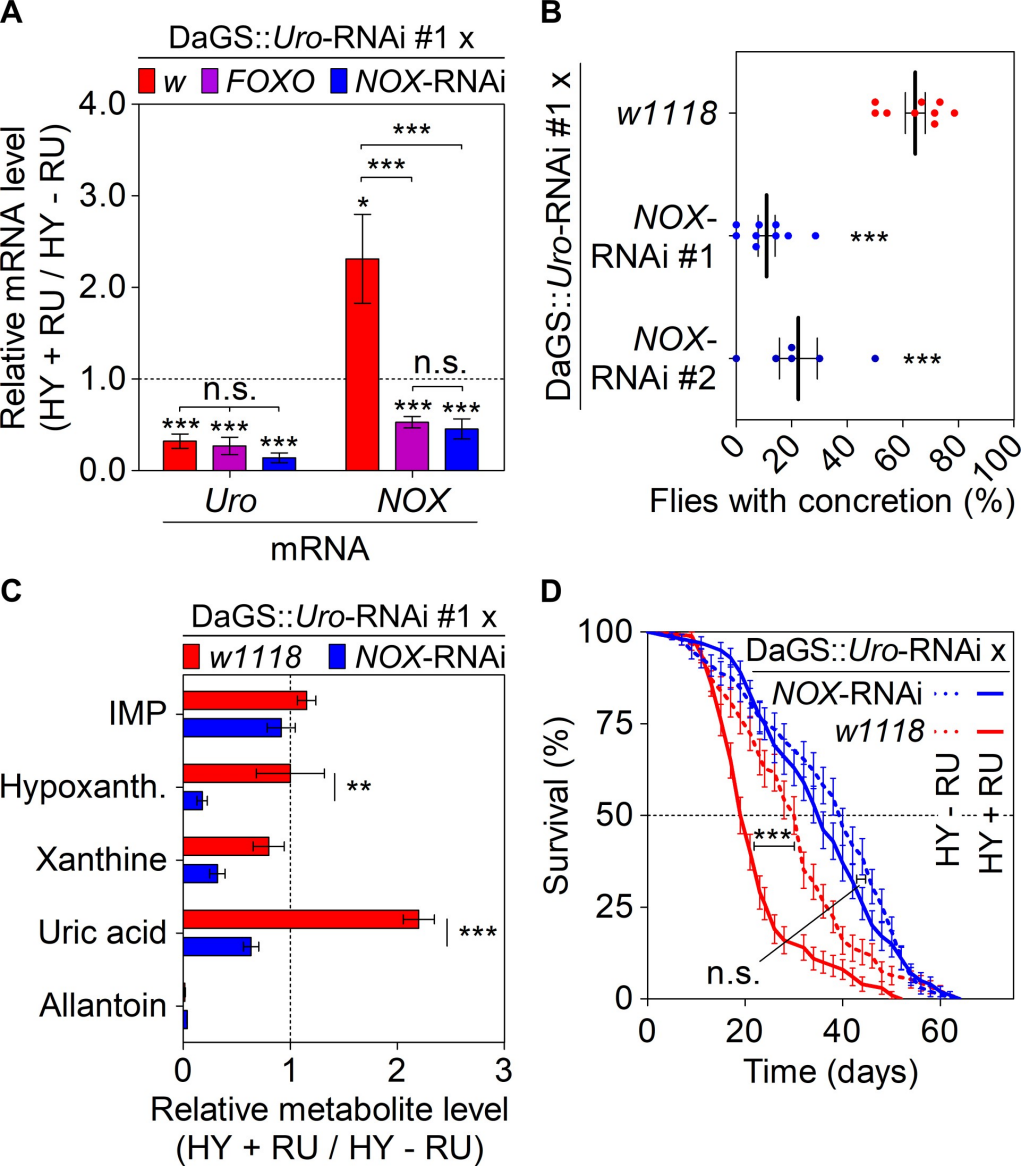
NOX mediated ROS production triggers lifespan attenuation and UA concretion formation on a high yeast diet. (A) Levels of *NOX* mRNA upon modulation of *FOXO*. Recombinant DaGS::*Uro*-RNAi #1 flies were crossed to *w1118* (*w*; no additional UAS-locus), or active UAS-transgenes triggering either over-expression of *FOXO* or inhibition of *NOX* (*NOX*-RNAi) and fed the HY + RU or HY - RU diet before relative mRNA levels of *urate oxidase* (*Uro*) and *NADPH oxidase* (*NOX*) were determined by qRT-PCR. (B) Concretion formation of recombinant DaGS::*Uro*-RNAi #1 flies crossed to *w1118* or one of the two *NOX*-RNAi lines after 14 days of feeding the HY + RU diet. (C) Relative purine metabolite levels of fly homogenates from recombinant DaGS::*Uro*-RNAi #1 flies crossed to *w1118* or *NOX*-RNAi #1. Metabolite levels were compared after 14 days of feeding flies the HY + RU or HY - RU diet. (D) Survival curves of flies from (C). Error bars represent the SE of multiple biological repeats. Supporting data is shown in S4 Fig and S5 Fig.

The increase of ROS levels upon *Uro* knockdown (Fig 5D–5F) could stem from two sources: (1) inefficient expression of ROS combating genes, or (2) an overabundance of ROS-producing enzymes. From the eleven oxidative stress-related genes whose expression was examined in the *Uro* knockdown flies, only the ROS-producing *NADPH Oxidase* (*NOX*) showed a twofold higher mRNA content (Fig 6A, S4E Fig). Elevated *NOX* expression was reversed completely by either *FOXO* over-expression or by introducing a *NOX*-RNAi element into DaGS::*Uro*-RNAi flies (Fig 6A, *NOX* mRNA). Of note, neither the *FOXO* over-expression nor the presence of the *NOX*-RNAi element altered efficiency of the *Uro* knockdown (Fig 6A, *Uro* mRNA). Next, we asked if reduction of *NOX* expression via RNAi could alleviate the concretion formation characteristically observed in *Uro* knockdown flies. As with *FOXO* over-expression, knocking down *NOX* expression in *Uro* knockdown flies significantly reduced UA accumulation, which was demonstrated with two different *NOX*-RNAi lines (Fig 6B). Further comparison of purine metabolite levels between *Uro* knockdown flies (DaGS::*Uro*-RNAi x *w1118*) and the *Uro* plus *NOX* double knockdown flies (DaGS::*Uro*-RNAi x *NOX*-RNAi) identified a significant drop of UA levels and depicted the interwoven nature of the NADPH oxidase with purine catabolism (Fig 6C). We than speculated whether the effect of NOX depletion extended to an improvement in lifespan. Like concretion formation, the recombinant DaGS::*Uro*-RNAi line also recapitulated the lifespan attenuation on a HY diet when crossed to *w1118* (Fig 6D, S5A Fig) or the mCherry-RNAi strain b35785 (S5B Fig). The median lifespan of DaGS::*Uro*-RNAi x *w1118* flies was reduced by 35% from 31 days without RU486 to 20 days in presence of RU486 (Fig 6D). This lifespan attenuation on the HY diet was partially rescued in DaGS::*Uro*-RNAi x *NOX*-RNAi flies using *NOX*-RNAi #1 or #2 (Fig 6D, S5F Fig).

**Fig 7:**
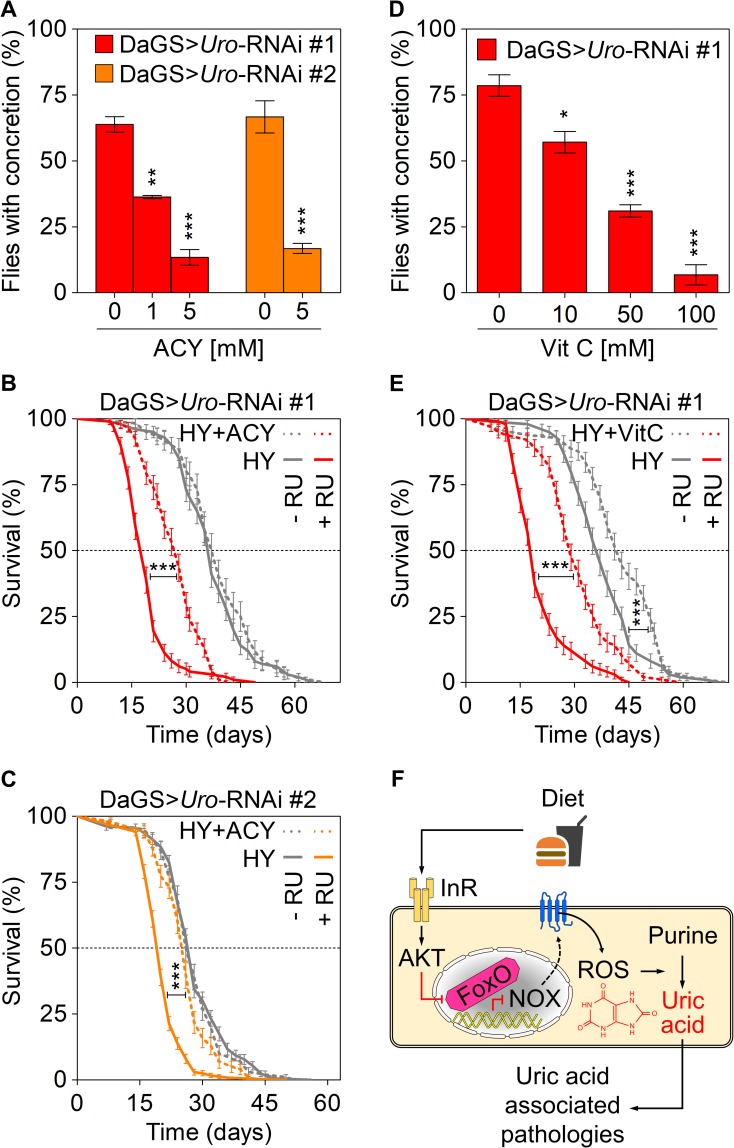
Pharmacological NOX inhibition phenocopies its genetic ablation and reduces UA related pathologies. (A) Concretion formation of DaGS>*Uro*-RNAi #1 or DaGS>*Uro*-RNAi #2 flies after 14 days of feeding a HY + RU diet supplemented with the indicated concentration of the NOX inhibitor apocynin (ACY). (B, C) Survival curves of DaGS>*Uro*-RNAi #1 (B) or DaGS>*Uro*-RNAi #2 (C) flies fed the HY diet without or with 5 mM ACY (+ACY) supplementation in presence or absence of RU. (D) Concretion formation of DaGS>*Uro*-RNAi #1 flies after 14 days of feeding a HY + RU diet supplemented with the indicated concentration of the ROS scavenger vitamin C (Vit C). (E) As in (B), but 50 mM Vit C (+VitC) supplementation. (F) Key players influencing UA levels and associated pathologies are summarized. See text for more details. Error bars represent the SE of multiple biological repeats.

To corroborate the *NOX* related findings, we used a pharmacological approach by inhibiting NOX activity and the associated ROS production using either apocynin (ACY; a NOX inhibitor) or vitamin C (Vit C; a ROS scavenger), respectively. Dietary ACY supplementation reduced the concretion formation of DaGS>*Uro*-RNAi flies on the HY plus RU486 diet in a dose-dependent manner. Concretion formation was reduced for both *Uro*-RNAi fly populations from about 70% without ACY supplementation down to 15% in presence of 5 mM ACY (Fig 7A). In terms of lifespan, a 5 mM ACY supplementation to the HY diet was able to extend the short lifespan of both *Uro* knockdown fly lines whereas the corresponding control siblings (DaGS>*Uro*-RNAi #1 / #2 without RU486) showed no change of survival in presence of ACY (Fig 7B and 7C). Thus, the ACY treatment very much mirrored the results obtained with the *NOX*-RNAi (cf. Figs 6B, 6D and 7A–7C). Last, we speculated if a water-soluble, i.e. membrane-permeable, ROS scavenger like Vit C could reduce the elevated ROS level found in *Uro* knockdown flies. Supplementation of the HY diet with increasing amounts of Vit C ranging from 10 to 100 mM gradually reduced concretion formation of *Uro* knockdown flies (Fig 7D). 50 mM Vit C addition to the HY diet also rescued the lifespan effect observed in flies with reduced urate oxidase levels (Fig 7E). Like the MTX treatment (Fig 4E), dietary Vit C supplementation also extended the median survival of control flies (Fig 7E). Worth mentioning, foods rich in Vit C are associated with a reduced risk for elevated UA and gout, due to its uricosuric effect [66, 67]. While Vit C seemed to extend the median lifespan of both the control (DaGS>*Uro*-RNAi #1 without RU486) and *Uro* knockdown flies (DaGS>*Uro*-RNAi #1 with RU486) by 16% and 53%, respectively, the impact of apocynin on lifespan was specific for the urate oxidase depleted animals (Fig 7B, 7C and 7E). In presence of RU486, ACY extended the median lifespan of DaGS>*Uro*-RNAi #1 flies by 47% (Fig 7B) and that of DaGS>*Uro*-RNAi #2 flies by 30% (Fig 7C).

In summary, our study argues the importance of purines as a dietary component that limits lifespan. Generally, mutants in genes that influence lifespan upon dietary restriction either extend lifespan upon rich nutrient conditions while failing to extend lifespan upon dietary restriction conditions or attenuate the maximum lifespan upon dietary restriction. However, *Uro* mutants belong to a “novel” class of genes that limit lifespan only upon rich conditions but have little or no influence upon dietary restriction. We hypothesize that mutants that display such a phenotype encode a gene that amplifies the cellular damage that takes place under rich nutrient conditions compared to dietary restriction. We speculate that the increase of UA is deleterious, and a lower UA level is partially responsible for the lifespan extension upon dietary restriction. The fly model established here will also be useful in dissecting the pathological effects of elevated UA on crystal formation as well as the underlying genetic factors driving crystal formation in the first place. The latter point is of particular interest, considering that only 5% of hyperuricemic people with SUA above 9 mg/dl develop gout [8, 9]. The genetic predisposition influencing UA accumulation is currently subject of genome-wide association studies and is complemented by our approach combining *in vivo* fly work and human candidate gene studies. Our data showing that genes such as *AKT*, *FOXO* and *NOX* are associated with UA levels and the incidence for deposit formation start shedding light on this important issue. In addition, the fly model will be beneficial to study the larger role of UA and UA deposits on influencing mortality as was underpinned by recent studies linking exceptional longevity and a reduced prevalence of age-related diseases with UA levels at the lower end of the human SUA spectrum [21, 22].

Our work demonstrates that inhibition of *Uro* in flies can serve as a useful model to study UA induced pathologies related to lifespan and crystal formation. Our results also highlight the importance of ILS signaling, and its downstream effector NOX, as an important mediator of UA metabolism and related pathologies (Fig 7F). Future research using this model can help determine the role of the several candidate genes in humans that have been implicated in altering UA levels. Given the over-nutrition encountered in developed countries, the causal nexus uncovered here identifies potential new drug targets that could help regulate UA levels, ameliorating pathologies associated with hyperuricemia and extending human healthspan.

## Material and methods

### Contact for reagent and resource sharing

Further information and requests for resources and reagents should be directed to and will be fulfilled by the Lead Contact, Pankaj Kapahi (pkapahi@buckinstitute.org).

### Experimental model and subject details

#### Fly strains used in this study

Fly strains purchased from Bloomington Drosophila Stock Center are indicated by the prefix “b”, strains from FlyORF, Zurich by “F”, strains from NIG-Fly, Mishima by “n”, and strains from VDRC, Vienna by “v”.

*GAL4* driver lines: *daughterless* gene switch (kindly provided by Linda Partridge), *daughterless* (b55851), *c42* (b30835), *Uro* (b44416), *Elav* (b458)

*UAS* responder lines: *Uro*-RNAi #1 (n7171-R1), *Uro*-RNAi #2 (v330484), *InR*-RNAi (b31594), *InR*-DN (b8252), *PTEN* (kindly provided by Tiang Xu), *AKT*-RNAi (b31701), *FOXO* #1(b9575), *FOXO* #2 (F000143), *Rel*-RNAi (b28943), *Dif*-RNAi (b29514), *Dl*-RNAi (b32934), *NOX*-RNAi #1 (b32902), *NOX*-RNAi #2 (b32433), *GAL4*-RNAi (b35784), *PRPS*-RNAi (b35619), *DHFR*-RNAi (b35015), *XDH*-RNAi (v25175)

Other lines: *w1118*, b35785 (expresses a dsRNA for RNAi of mCherry under UAS control). We generated a recombinant fly line carrying both genetic elements (+/+; DaGS::*Uro*-RNAi #1/ DaGS::*Uro*-RNAi #1; +/+) on chromosome two. This line was crossed to UAS responder lines to introduce an additional transgenic element (including the no target UAS-mCherry-RNAi line b35785) or *w1118* (Fig 4C, Fig 5A, 5C, 5E and 5F, Fig 6 and S4C, S4D and S5 Figs).

With the exception of *Uro*-RNAi #2 (v330484) all other fly strains were routinely outcrossed into the commonly used *w1118* background to achieve genetic homogeneity amongst different strains and provide isogenization.

#### Fly husbandry, dietary and pharmacological manipulations

All fly lines were maintained on standard fly yeast extract medium containing 1.5% yeast, 5% sucrose, 0.46% agar, 8.5% of corn meal, and 1% acid mix (a 1:1 mix of 10% propionic acid and 83.6% orthophosphoric acid) prepared in distilled water. To prepare the media, cornmeal (85 g), sucrose (50 g), active dry yeast (16 g, "Saf-instant") and agar (4.6 g) were mixed in a liter of water and brought to boil under constant stirring. Once cooled down to 60°C 10 ml of acid mix was added to the media. The media were then poured in vials (~10 ml/ vial) or bottles (50 ml/bottle) and allowed to cool down before storing at 4°C for later usage. These vials or bottles were then seeded with some live yeast just before the flies are transferred and used for maintenance of lab stocks, collection of virgins, or setting up crosses.

Experimental low and high yeast diets varied only with regard to the yeast type and content ranging from 0% to 8%. Diets declared as LY (low yeast content) and HY (high yeast content) contained 0.5% and 5% Baker's yeast extract (212750 Bacto Yeast Extract, B.D. Diagnostic Systems, Sparks, MD), respectively. If not stated otherwise pharmacological treatments or dietary supplementations were mixed with the other ingredients during preparation of the food. As for the standard medium, the mix was brought to a boil under constant stirring and allowed to cool down to 60°C before adding the acid mix. For the induction of transgenic elements via the *GAL4-UAS* system when using a gene switch driver 200 μM RU486 (Mifepristone, Sigma) in 100% ethanol was added to the food while the isogenic controls received vehicle treatment. The media was then poured in vials (~5 ml/vial) and allowed to cool down before storing at 4°C for later usage. Further dietary supplementations and drug treatments used in our study are summarized in Table 2.

**Table 2:** Dietary supplementations and drug treatments.

Dietary Additive	Ingredients	Vendor	Concentrations
10 mM Pu (Purines)	Adenine, Guanine	Sigma-Aldrich	5 mM each
20 mM Pu	Adenine, Guanine	Sigma-Aldrich	10 mM each
40 mM Pu	Adenine, Guanine	Sigma-Aldrich	20 mM each
20 mM Py (Pyrimidines)	Cytosine, Thymine	Sigma-Aldrich	10 mM each
AP	Allopurinol	Sigma-Aldrich	1, 5, 10 mM
MTX	Methotrexate	Sigma-Aldrich	0.2, 1, 5, 50 μM
Vit C	Vitamin C	Sigma-Aldrich	10, 50, 100 mM
ACY	Apocynin	Sigma-Aldrich	1, 5 mM
5% Protein	BSA, Casein	Cell Signaling Technology, Sigma-Aldrich	2.5% (w/v) each
15% Protein	BSA, Casein	s. above	7.5% (w/v) each
10% Sucrose	Sucrose	Mallinckrodt Chemicals	10% (w/v)
20% Sucrose	Sucrose	s. above	20% (w/v)

#### Fly rearing

Genetic crosses were obtained by pairing 15 young virgin females (carrying the *GAL4* driver construct) with 4 young male flies (carrying the *UAS* responder construct) in new stock bottles. Flies were kept in the stock bottles for five days, after which the parents were removed, and the larvae could develop in a temperature (25°C) and humidity (60%) controlled designated fly room with a 12-hour light / dark cycle. Newly eclosed flies could mate for 2–3 days, to complete development post-eclosion, before they were grouped under light CO_2_ anesthesia. Sorted females were then transferred to the appropriate media vials for subsequent analyses.

### Method details

#### Lifespan analysis

Flies developed on standard fly 1.5% yeast extract medium were transferred to the necessary diet within 72 hours after eclosion. For survivorship analysis four to six media containing vials with 25 mated females were kept in a temperature (25°C) and humidity (60%) controlled designated fly room with a 12-hour light / dark cycle. Every other day flies were transferred to fresh food and fly survival was scored by counting the number of dead flies. The significance of change was determined using the log-rank test. Each lifespan was repeated at least once to generate independent, biological replicates.

#### Dissection and scoring of concretion formation

For dissection and imaging assays adult female flies were dissected after 4, 7, 11, or 14 days. The excretory system, in particular the hindgut and Malpighian tubule area was checked and imaged for the presence of ectopic biomineralization. Flies were anesthetized by CO_2_ on standard flypads (Cat # 59–108, Genesee Scientific, San Diego, CA) and dissected under a dissecting light microscope (SZ61, Olympus, Center Valley, PA) on a culture dish in droplets of distilled water utilizing fine forceps (Roboz ceramic, Gaithersburg, MD). The excretory system was imaged utilizing a Zeiss Stereo Microscope with external light source. While transparent posterior midguts and hindguts devoid of any sign of deposit formation were scored as negative, those with pale yellow/orange hard concretions were scored as concretion carrying specimens. The relative level of concretion formation was quantified by this binary approach. Per condition at least 15–20 flies were dissected and the number of flies with deposits was divided by the total number of flies dissected. Each experiment was repeated for a minimum of three independent, biological replicates.

#### Imaging of ectopic biomineral

Images of dissected guts and Malpighian tubules were captured with the Olympus SZX12 microscope. Isolated deposits extracted from the hindgut area were imaged with the Olympus BX51 microscope (Olympus Scientific Solutions Americas Corp. Waltham, MA).

For the micro-computer tomography (μCT) imaging fly specimens were scanned in 50% ethanol using a micro-XCT unit at 4X magnification (4.5 μm/voxel) with 1200 image projections. Scanning was performed with a 3 second exposure, source power of 40 W, 200 μA current, source distance of ~30 mm, detector distance of ~14 mm, camera binning 2, and an angle sweep from -93° to 93°. Post-3D reconstruction, the acquired data was further processed and analyzed with Avizo software (Version 9.3.0; FEI, Hillsboro, Oregon).

#### Collection of deposits for mass spectrometry-based metabolomics

Flies were dissected in 50% methanol/water mix and the deposits from the hindgut area were isolated using fine forceps. Deposits of 25 flies carrying concretions were collected in an Eppendorf tube filled with 500 μl 50% methanol/water. The material was sedimented by centrifugation for 15 s with 5,000 rpm and the supernatant was aspirated. After two further wash steps with 500 μl 50% methanol/water deposits were frozen in liquid nitrogen and stored at -80°C until further processing.

#### High-performance liquid chromatography (HPLC) mass spectrometry (MS)

High-performance liquid chromatography (HPLC) was performed using an Agilent 1260 UHPLC system and connected to a Phenomenex Luna NH2 column (2 x 100 mm, 3 μm, 100 Å) and a SecurityGuard NH2 guard column 4 x 2 mm ID. Mass spectrometry (MS) was performed using a 5500 Triple-Quadrupole LC-MS/MS mass spectrometer from Sciex fitted with a Turbo VTM ion source. Sciex’s Analyst v1.6.1 [68] was used for all forms of data acquisition, development of HPLC method, and optimization of analyte-specific MRM (multiple reaction monitoring) transitions. Skyline v4.1 was used for LC-MS/MS data analysis. For whole fly analysis, 5 flies (in sextuplicates) per condition were flash-frozen over liquid nitrogen and subsequently homogenized ultrasonically using a Fisher Scientific’s 550 Sonic Dismembrator with 50 μl of an 8:2 mixture of methanol/water (v/v), containing 2.5 μM of 2-chloroadenosine as internal standard. Three 20 s pulses at amplitude setting 3 of the instrument (on ice) were sufficient to completely homogenize fly specimens. The homogenates were then vortexed for 5 times over a period of ~30 min (each 1 min long). Subsequently, the samples were centrifuged at 10,000 rpm for 10 min, the supernatant was filtered, and 3 μl of each was injected for HPLC-MRM analysis (*vide infra*) without any additional processing. For the analysis of fly deposits, samples were collected as described above and prepared for HPLC-MRM analysis (*vide infra*) as described above for whole flies.

Optimization of analyte-specific MRM transitions, such as determination of suitable precursor and product ions and optimal MS parameters for each transition (Q1, precursor → Q3, product) were achieved by isocratic flow injection of the 1–10 μM solution (final) for each standard, diluted in 80% methanol. The most intense transition was used as quantifier, whereas one or more additional transitions were used as qualifier for each compound (S3 Table). A final standard mixture of all compounds at 5 μM (containing the internal standard 2-chloroadenosine at 2.5 μM), were prepared prior to analysis and injected at the onset of each biological sample set.

Based on previous reports [69], the following HPLC program was developed: a solvent gradient of 20 mM ammmonium acetate + 20 mM ammonium hydroxide (pH = ~9.5) + 5% acetonitrile (aqueous)–acetonitrile (organic) was used with 0.4 ml/min flow rate, starting with an acetonitrile content of 85% for 1 min, which was decreased to 30% over 3 min and then to 0% over 7 min and held at 0% for 2 min. The HPLC column was subsequently reconstituted to its initial condition (acetonitrile content of 85%) over the next 1 min and re-equilibrated for 7 min. Metabolome extracts from whole flies or deposits were analyzed by HPLC-MRM with positive/negative switching of source ion modes [69]. Source conditions were as follows: curtain gas (CUR) 20, collision gas (CAD) 7, ion source gas 1 (GS1) 30, ion source gas 2 (GS2) 30, ionspray voltage (IS) ±4500 V, and source temperature (TEM) 450°C. Quantification was based on integration of analyte-specific peaks obtained from HPLC-MRM runs.

#### Feeding assay

After 14 days of feeding a particular diet, flies were switched to blue dye medium (15% sucrose, 1% agar, 1% FD&C Blue #1) for 2 hours. Flies were then frozen in liquid nitrogen. To prevent eye pigment from interfering with absorbance spectrum, heads were separated from the bodies and bodies were then homogenized in PBS buffer and centrifuged at 13,000 rpm for 25 minutes. Supernatants were transferred to a 96-well plate and analyzed for absorbance at 625 nm with use of SpectraMax M2 spectrophotometer [70].

#### Dihydroethidium (DHE) staining for ROS measurements

Intact guts with adhering Malpighian tubules were dissected from female flies in a droplet of PBS (137 mM NaCl, 2.7 mM KCl, 10 mM Phosphate, pH 7.4) on ice and subsequently stained for 5 min with 45 μM DHE (Thermo Fisher Scientific) dissolved in DMSO (Sigma Aldrich). After three 10 min wash steps with PBS the sample was fixed for 45 min using 4% formaldehyde (Sigma Aldrich) in PBS followed by another three 10 min wash steps. After a 15 min staining with 1 μg/ml DAPI (Thermo Fisher Scientific) dissolved in PBS the specimen was mounted onto a microscope slide using Mowiol (Sigma Aldrich). Confocal images were collected using a Zeiss LSM700 confocal system using an Excitation/Emission (nm) of 518/605. DHE intensity was quantified using Image J.

#### Total RNA and cDNA preparation

Total RNA was isolated from 5–10 females using the Quick-RNA MiniPrep Kit (Zymo Research) at room temperature. In brief, flies were anesthetized with CO_2_ before homogenization using a Kontes Microtube Pellet Pestle Rods with Motor in an Eppendorf tube containing 200 μl RNA Lysis Buffer. After adding another 400 μl RNA Lysis Buffer the homogenate was centrifuged for 1 min with 12.000 rcf and 400 μl supernatant was transferred to the spin-away filter placed over a fresh collection tube for elimination of genomic DNA. 400 μl of 95% ethanol was added to the flow through and mixed thoroughly. Transfer mixture to Zymo-Spin IIICG column in a collection tube and centrifuge for 1 min with 12,000 rcf. After adding 400 μl RNA Prep Buffer and a 30 sec centrifugation with 12,000 rcf two more wash steps with 700 μl and 400 μl RNA Wash Buffer follow. To ensure complete removal of the RNA Wash Buffer the last centrifugation is carried for 2 min with 12,000 rcf. The Zymo-Spin IIICG column is placed in a collection tube and bound RNA is eluted with 30 μl DNAse/RNAse-free water in a collection tube by centrifugation for 30 sec with 12,000 rcf. Quantity and quality of isolated RNA was determined using the NanoDrop 1000 Spectrophotometer (Thermo Scientific).

1 μg of total RNA in a volume of 16 μl was used per sample and cDNA was synthesized using 4 μl iScript Reverse Transcription Supermix for RT-qPCR (Bio-Rad) according to the manufacturer’s protocol. The RT-PCR reaction protocol included a priming step (5 min at 25°C) followed by the reverse transcription (30 min at 42°C) and inactivation of the reaction (5 min at 85°C). If necessary, cDNA was stored at -20°C before the quantitative real-time PCR.

#### Quantitative real-time PCR (qRT-PCR)

Using 3 μl of the 1:10 diluted cDNA as a template, 2 μl of a primer pair (500 nM each) and 5 μl SensiFAST SYBR No-ROX Kit (BIOLINE) the qPCR was performed using the Light Cycler 480 Real-Time PCR System (Roche Applied Science). After pre-incubating the sample (95°C for 2 min) to denature DNA forty PCR cycles of denaturing (95°C for 5 s, ramp rate 4.8°C/s), as well as annealing and extension (60°C for 20 s, ramp rate 2.5°C/s) followed. The specificity of amplicons was verified with a subsequent melting curve analysis. The data was analyzed by means of the ΔΔCt method and the values were normalized using β-tubulin as an internal control. The primer pairs used in this study are summarized in Table 3.

**Table 3:** Primer pairs used for the qRT-PCR.

Gene name	Forward Primer Sequence	Reverse Primer Sequence
CG9277 (*β-Tubulin*)	acatcccgccccgtggtc	agaaagccttgcgcctgaaca
CG7171 (*Uro*)	gcgatgtggttataaggagaaca	tcttcagcacccggagac
CG3143 (*FOXO*)	cgagagtccgctccacag	aagatcctgcgccctaatg
CG34399 (*NOX*)	ccttccgcaagctattcct	tcgagatcaaacagctggaa
CG9127 (*Ade2*)	cgactgcgtgatgtccag	gctcgtacggctgtttgtaac
CG3989 (*Ade5*)	ccaccacaacagcatccata	gctgaggagcaggcaaag
CG4006 (*AKT*)	atgacgccatctgaacagac	cttctcgcgacacaaaataacc
CG18402 (*InR*)	acctatttaaccacaagcga	ctcgatagttccaagattgc
CG8846 (*Thor*)	cccacttcccttttatctctctc	acagaccgcaattgtcctg
CG18315 (*Aprt*)	gattcgcaagaagggcaag	tttgcagctcaaaggtgtca
CG4827 (*Veil*)	ggtgggtcagtcagttagcc	gaccacgggcaggtaacat
CG10146 (*Attacin A*)	cacaatgtggtgggtcagg	ggcaccatgaccagcatt
CG12763 (*Diptericin*)	cacgagattggactgaatgg	tttccagctcggttctgagt
CG8175 (*Metchnikowin*)	tcttggagcgatttttctgg	tctgccagcactgatgtagc
CG6871 (*Catalase*)	tgactacaaaaactcccaaacg	ttgattccaatgggtgctc
CG3131 (*DUOX*)	agccctgctgcttctactga	cgctgtttctcggtctgact
CG5164 (*Gst-E1*)	ggactacgagtacaaggaggtga	tcacatattcctcgctcaggt
CG12013 (*PHGPx*)	ttacgcttcgaaagtgagaaagt	cgttcttgtaatctccgttagca
CG5826 (*Prx3*)	gaagactacaggggcaagtacc	ggggcaaacgaatgtgaa
CG11793 (*SOD1*)	caagggcacggttttcttc	cctcaccggagaccttcac
CG8905 (*SOD2*)	aatttcgcaaactgcaagc	tgatgcagctccatgatctc
CG31884 (*Trx2*)	acatggtgtaccaggtgaaagat	caagtggcgaagaaatcca
CG2151 (*Trxr-1*)	gatgagcaccaaaggaggat	gaaatccagacaggccacac
CG7642 (*XDH*)	tggtgacttcccactggag	cctggttcgggtatttcaag

#### Human candidate-gene study (CGS)

Data from the Kaiser Permanente Epidemiology Research on Adult Health and Aging (GERA) Cohort, a subset of the Research Program on Genes, Environment and Health (RPGEH), were gathered for analysis. Detailed methods on the genotyping, imputation, and ancestry calculations can be found in [50]. The study population consisted of 80,795 adult subjects of European ancestry. The average age of the cohort was approximately 63 years old at the time of sample collection for genotyping. Two phenotypes were used for this study: (1) Gout, defined as two ICD-9 codes for gout in the electronic health record; (2) mean serum UA levels. Association analyses were conducted using linear or logistic regression models in PLINK v1.90 adjusting for population structure using the top 10 principal components. In the model, concomitant diuretics and gender were included as covariates because of their strong correlation with all phenotypes. Maximum body mass index (BMI) correlated strongly with UA levels, and was therefore also included as a covariate in all analyses.

SNPs within +/- 2kb of ILS genes (*FOXO1*, *FOXO3*, *AKT1*, *AKT2*, *AKT3*, *PTEN*) were analyzed. Lists of SNPs within genes were analyzed using NIH LDLink SNPclip tool [71], where SNPs in high linkage disequilibrium (R^2^>0.5) were pruned. Gene-specific p-value thresholds were set as 0.05/(2 phenotypes*number of SNPs in each gene post-pruning). As a quality control step, top associated SNPs were checked for extreme HWE departures in our samples.

R v3.4.0 was used to create phenotypes and analyze associations between risk factors and phenotypes.

### Quantification and statistical analysis

Bar graphs and scatter blots represent the mean ± standard error (SE), when indicated. Lifespan curves, and other graphs were visualized using GraphPad Prism 5 software. For statistical comparisons between the control and single treatment group a two-tailed student’s t-test was used; except for lifespan analysis where the log-rank test was performed. When multiple conditions were compared ANOVA was used followed by Tukey’s multiple comparison post-test comparing all pairs of conditions. Significant differences are indicated according to the p-value by asterisks with p < 0.001 (***) < 0.01 (**) < 0.05 (*). Non significant differences are indicated by n.s.

## Supporting information

S1 Fig *Uro* knockdown attenuates *Drosophila* lifespan specifically on diets with a high yeast extract content. Average median lifespan of DaGS>*Uro*-RNAi #1 flies fed diets with the indicated yeast concentration in absence (- RU) or presence (+ RU) of RU486. The average median lifespan was deduced from individual Kaplan-Meier survival curves and is defined as the time point in days when 50% of the population is alive. Error bars represent the SE of multiple biological repeats. Lifespans for the 2, 3, 4, and 8% yeast concentration were not repeated.
(TIF)

S2 Fig *Uro* knockdown enhances UA concretion formation in the excretory system specifically on a HY diet. (**A**) Concretion formation of DaGS>*Uro*-RNAi #1 flies after 14 days of feeding a diet with the indicated yeast concentration supplemented without (- RU) or with (+ RU) RU486. (**B**) Concretion formation of DaGS>*Uro*-RNAi #2 flies reared on a HY - RU or HY + RU diet for 14 days. Error bars represent the SE of multiple biological repeats.
(TIF)

S3 Fig Overview of purine homeostasis, inhibitors thereof, and their impact on food consumption. (**A**) Shown are key enzymes (red), inhibitors (blue) thereof, and metabolites (black) of the tripartite purine metabolism orchestrated by purine *de novo* synthesis, salvage and degradation. (**B**) Using a colorimetric assay, the food intake of DaGS>*Uro*-RNAi #1 flies was compared after exposure to the indicated HY + RU diet supplemented without (-) or with 10 mM allopurinol (AP) or 50 μM methotrexate (MTX) for 14 days. AU = arbitrary units.
(TIF)

S4 Fig FoxO activity and antimicrobial peptide expression in *Uro* knockdown flies fed a high yeast diet. (**A**) Relative mRNA levels of transcripts encoding for FoxO target genes (taken from [56]) were determined by qRT-PCR. Genes in the orange and blue area were previously determined as targets up-regulated and down-regulated by induction of *FOXO* expression, respectively. Genes of interest belong to purine metabolism (*phosphoribosylformylglycinamidine synthase* (*Ade2)*, *PAICS bifunctional enzyme (Ade5)*, *adenine phosphoribosyltransferase* (*Aprt)*, *Veil*) or the ILS pathway (*AKT*, *InR*, *Thor*). DaGS>*Uro*-RNAi #1 flies were fed the high yeast diet with RU486 (HY + RU) or without RU486 (HY - RU) for 14 days before comparing gene expression levels in the *Uro* knockdown and control flies. (**B**) Relative mRNA levels of transcripts encoding for the antimicrobial peptides *attacin A* (*Att A*), *diptericin* (*Dipt*), and *metchnikowin* (*Metch*) were determined by qRT-PCR. DaGS>*Uro*-RNAi #1 or DaGS>*Uro*-RNAi #2 flies were fed the HY + RU or HY - RU diet for 14 days before comparing expression levels. (**C**) As in (**B**) the mRNA levels were determined by qRT-PCR from the recombinant DaGS::*Uro*-RNAi #1 flies crossed to *w1118* (no additional UAS-locus), or strains with active UAS-transgenes triggering either over-expression of *FOXO* (*FOXO* #1, *FOXO* #2) or inhibition of *NOX* (*NOX*-RNAi #1, *NOX*-RNAi #2). Flies were fed the HY + RU or HY - RU diet for 14 days. (**D**) The recombinant DaGS::*Uro*-RNAi #1 line was crossed to *w1118* (no additional UAS-locus), or active UAS-RNAi lines targeting one of the NFκB paralogs *Relish* (*Rel*-RNAi), *Dif* (*Dif*-RNAi) or *Dorsal* (*Dl*-RNAi). To measure concretion formation the flies were fed the HY + RU diet for 14 days prior to dissection. (**E**) Relative mRNA levels of transcripts encoding for indicated oxidative stress-related proteins were compared 4 and 14 days after feeding the HY + RU or HY - RU diet to DaGS>*Uro*-RNAi #1 flies. Error bars represent the SE.
(TIF)

S5 Fig NOX depletion rescues the short lifespan of *Uro* knockdown flies. (**A-E**) Survival curves of recombinant DaGS::*Uro*-RNAi #1 flies crossed to *w1118* (no additional UAS-locus), b35785 (carrying a no target UAS-mCherry-RNAi), or active UAS-RNAi lines targeting *NOX* (*NOX*-RNAi #1, *NOX*-RNAi #2) or the *GAL4* transcription factor (*GAL4*-RNAi). Flies were fed the transgene activating diet HY + RU or control diet HY—RU. (**F**) Average median lifespan from multiple repeats of the fly strains shown in **A-D** fed the different HY diets. Error bars represent the SE.
(TIF)

S1 Table Description of human candidate gene study (CGS) population. Serum uric acid (SUA) levels are not included in a typical doctors’ visit and are therefore missing for most healthy controls. Avg, average; max BMI, maximal body mass index in the evaluation period.
(DOCX)

S2 Table Top SNPs and associated effect sizes for each phenotype. SNPs reaching the gene-specific significance threshold are shown with their effect sizes and specific P-values. SNP, single nucleotide polymorphism; SE, standard error; OR, odds ratio; UA, uric acid.
(DOCX)

S3 Table Summary of the analyte-specific MRM (multiple reaction monitoring) transitions. (DOCX)
