# Supplementary figures and images for: A conserved role of the insulin-like signaling pathway in diet-dependent uric acid pathologies in Drosophila melanogaster
Source: PLoS Genet. 2019 Aug 15;15(8):e1008318. doi: 10.1371/journal.pgen.1008318 (PMC6695094; doi:10.1371/journal.pgen.1008318)

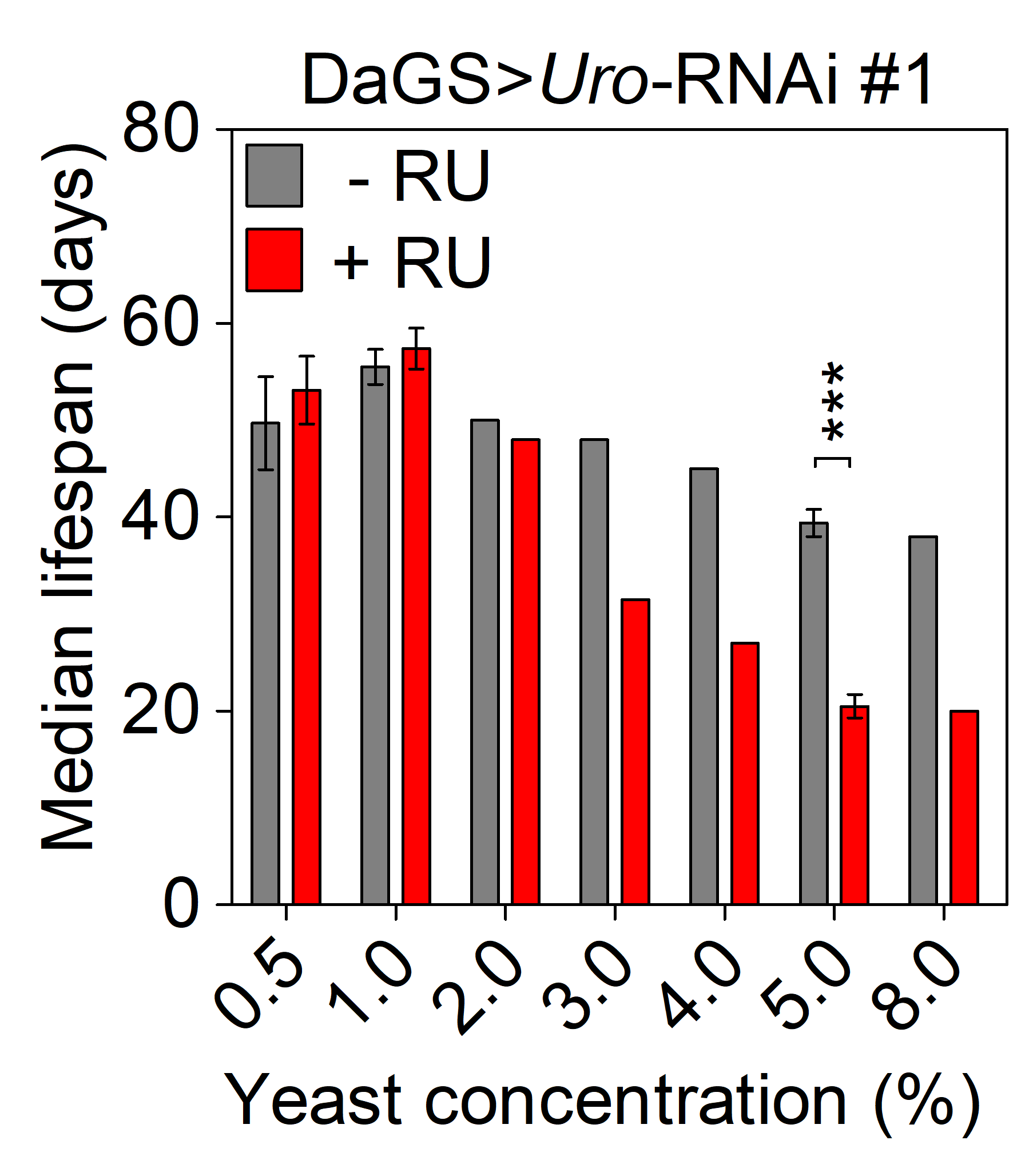

Supplement: S1 Fig — Average median lifespan of DaGS>Uro-RNAi #1 flies fed diets with the indicated yeast concentration in absence (- RU) or presence (+ RU) of RU486. The average median lifespan was deduced from individual Kaplan-Meier survival curves and is defined as the time point in days when 50% of the population is alive. Error bars represent the SE of multiple biological repeats. Lifespans for the 2, 3, 4, and 8% yeast concentration were not repeated. (TIF) [file pgen.1008318.s001.tif]

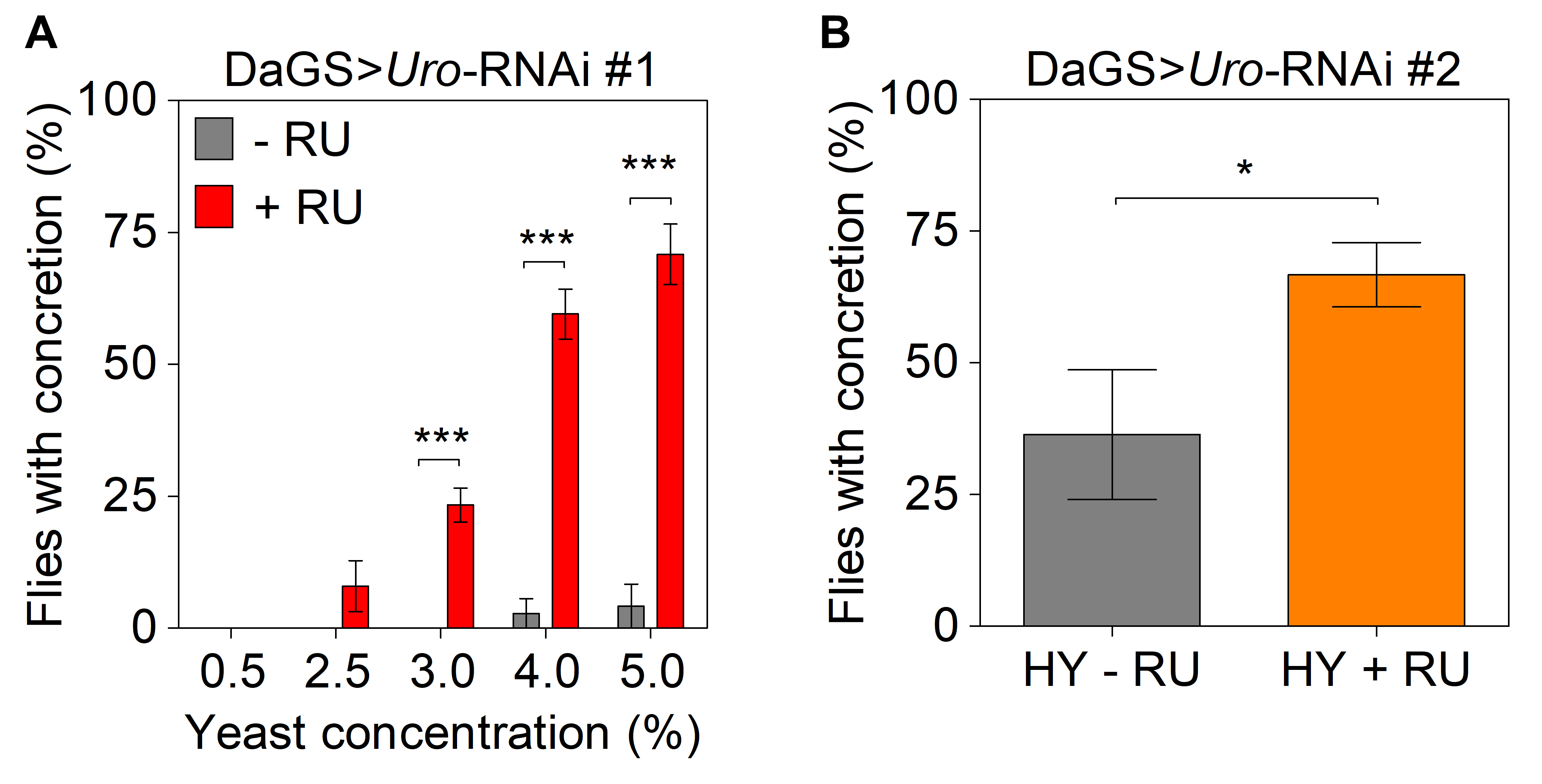

Supplement: S2 Fig — (A) Concretion formation of DaGS>Uro-RNAi #1 flies after 14 days of feeding a diet with the indicated yeast concentration supplemented without (- RU) or with (+ RU) RU486. (B) Concretion formation of DaGS>Uro-RNAi #2 flies reared on a HY - RU or HY + RU diet for 14 days. Error bars represent the SE of multiple biological repeats. (TIF) [file pgen.1008318.s002.tif]

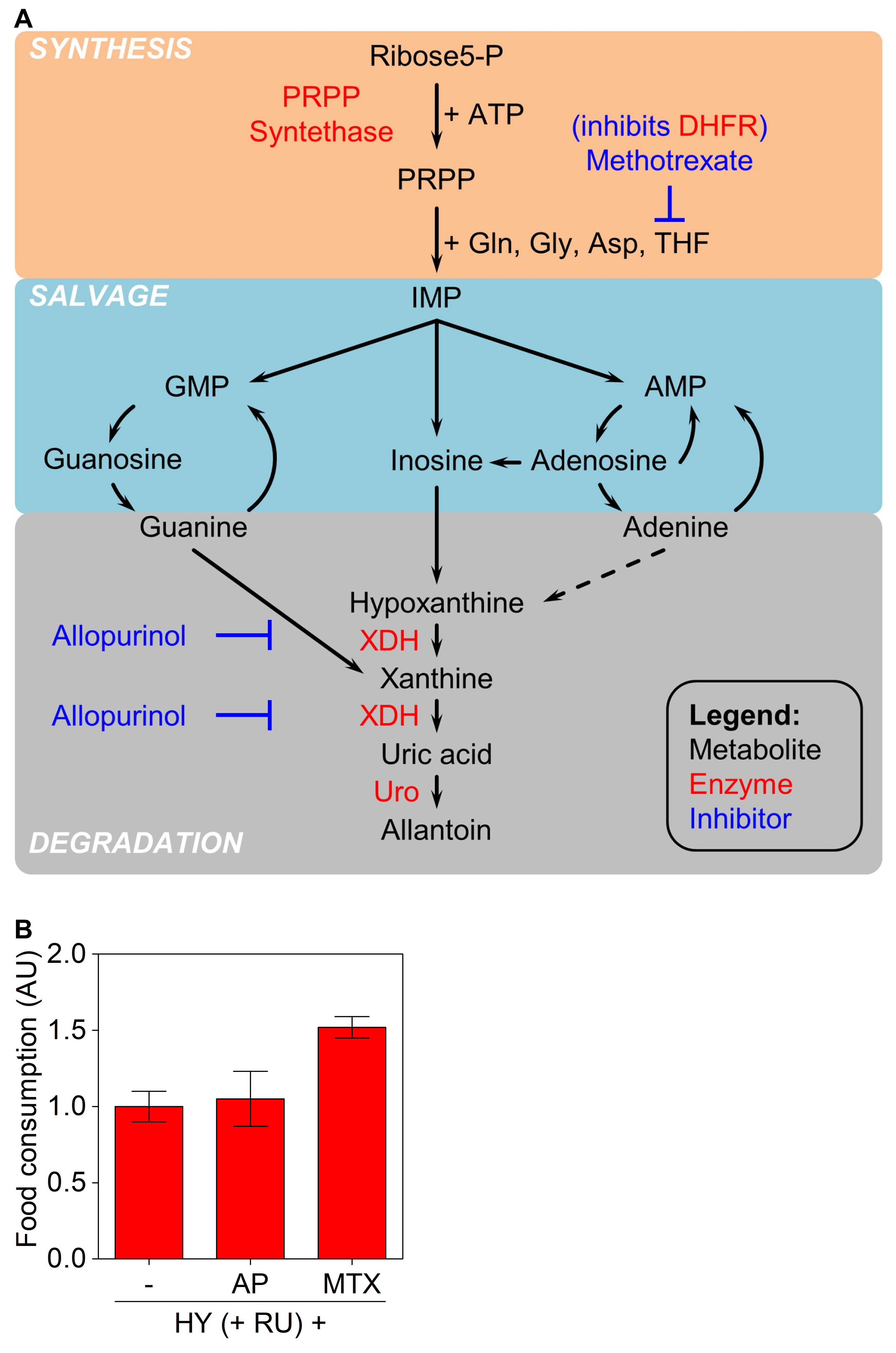

Supplement: S3 Fig — (A) Shown are key enzymes (red), inhibitors (blue) thereof, and metabolites (black) of the tripartite purine metabolism orchestrated by purine de novo synthesis, salvage and degradation. (B) Using a colorimetric assay, the food intake of DaGS>Uro-RNAi #1 flies was compared after exposure to the indicated HY + RU diet supplemented without (-) or with 10 mM allopurinol (AP) or 50 μM methotrexate (MTX) for 14 days. AU = arbitrary units. (TIF) [file pgen.1008318.s003.tif]

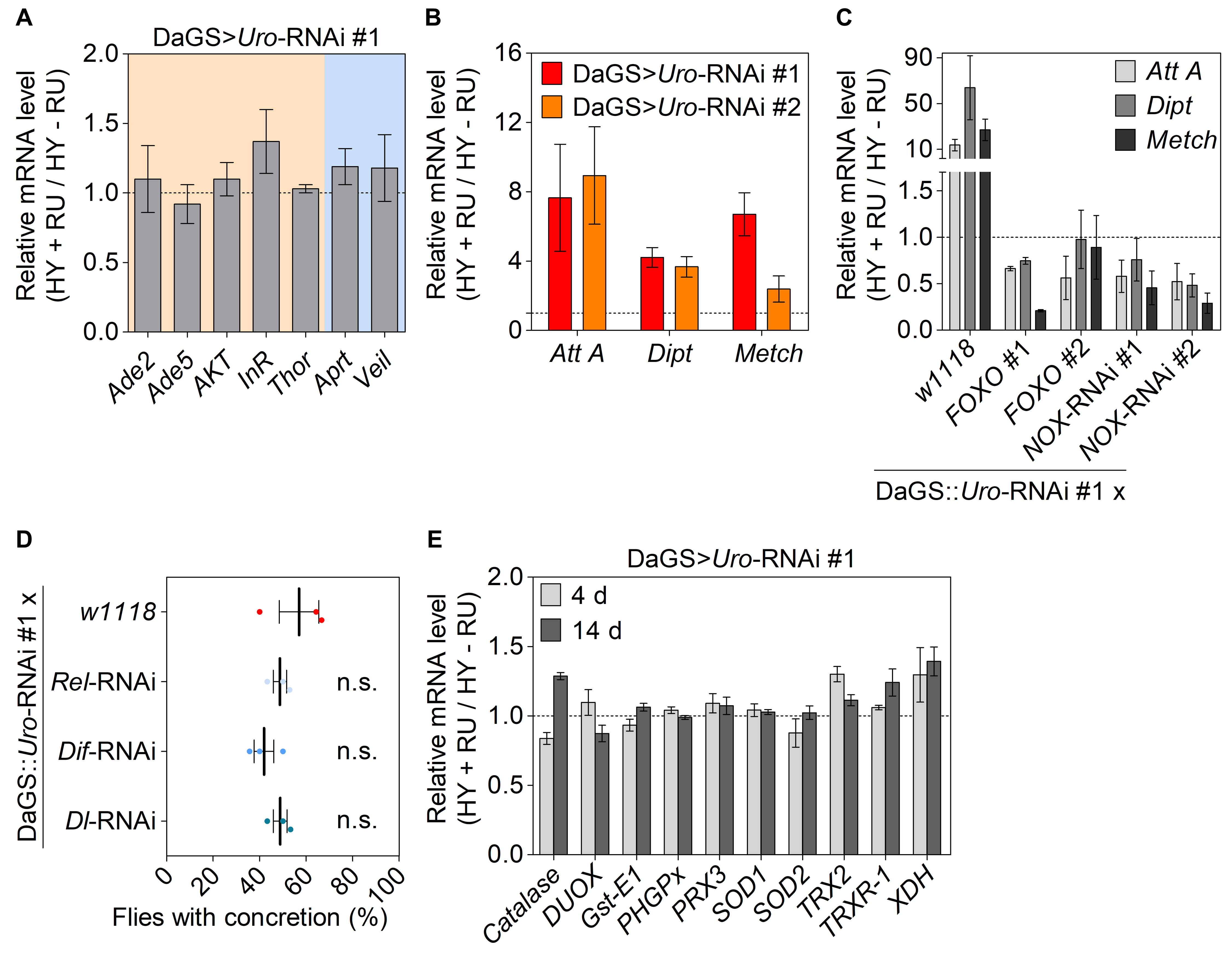

Supplement: S4 Fig — (A) Relative mRNA levels of transcripts encoding for FoxO target genes (taken from [56]) were determined by qRT-PCR. Genes in the orange and blue area were previously determined as targets up-regulated and down-regulated by induction of FOXO expression, respectively. Genes of interest belong to purine metabolism (phosphoribosylformylglycinamidine synthase (Ade2), PAICS bifunctional enzyme (Ade5), adenine phosphoribosyltransferase (Aprt), Veil) or the ILS pathway (AKT, InR, Thor). DaGS>Uro-RNAi #1 flies were fed the high yeast diet with RU486 (HY + RU) or without RU486 (HY - RU) for 14 days before comparing gene expression levels in the Uro knockdown and control flies. (B) Relative mRNA levels of transcripts encoding for the antimicrobial peptides attacin A (Att A), diptericin (Dipt), and metchnikowin (Metch) were determined by qRT-PCR. DaGS>Uro-RNAi #1 or DaGS>Uro-RNAi #2 flies were fed the HY + RU or HY - RU diet for 14 days before comparing expression levels. (C) As in (B) the mRNA levels were determined by qRT-PCR from the recombinant DaGS::Uro-RNAi #1 flies crossed to w1118 (no additional UAS-locus), or strains with active UAS-transgenes triggering either over-expression of FOXO (FOXO #1, FOXO #2) or inhibition of NOX (NOX-RNAi #1, NOX-RNAi #2). Flies were fed the HY + RU or HY - RU diet for 14 days. (D) The recombinant DaGS::Uro-RNAi #1 line was crossed to w1118 (no additional UAS-locus), or active UAS-RNAi lines targeting one of the NFκB paralogs Relish (Rel-RNAi), Dif (Dif-RNAi) or Dorsal (Dl-RNAi). To measure concretion formation the flies were fed the HY + RU diet for 14 days prior to dissection. (E) Relative mRNA levels of transcripts encoding for indicated oxidative stress-related proteins were compared 4 and 14 days after feeding the HY + RU or HY - RU diet to DaGS>Uro-RNAi #1 flies. Error bars represent the SE. (TIF) [file pgen.1008318.s004.tif]

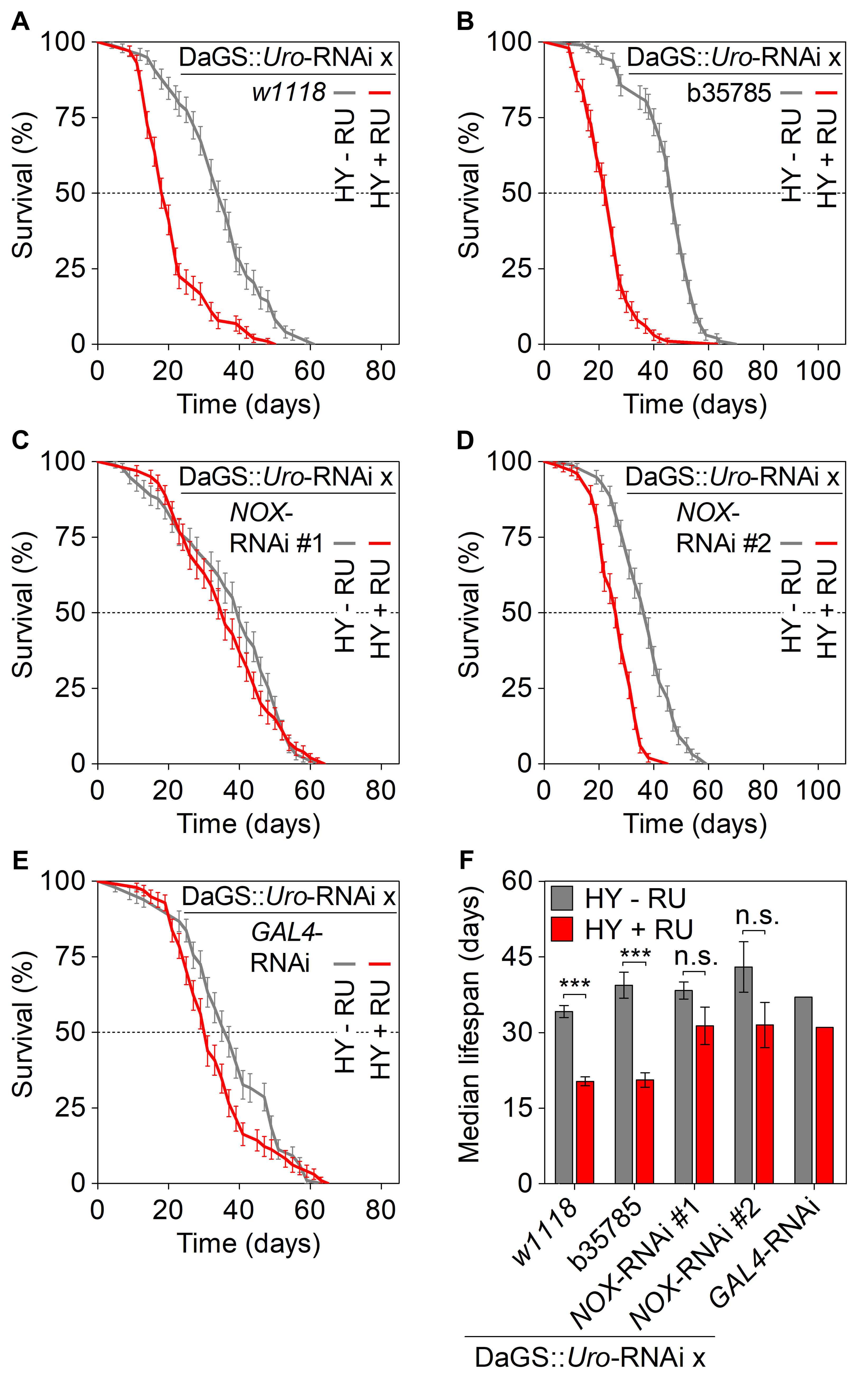

Supplement: S5 Fig — (A-E) Survival curves of recombinant DaGS::Uro-RNAi #1 flies crossed to w1118 (no additional UAS-locus), b35785 (carrying a no target UAS-mCherry-RNAi), or active UAS-RNAi lines targeting NOX (NOX-RNAi #1, NOX-RNAi #2) or the GAL4 transcription factor (GAL4-RNAi). Flies were fed the transgene activating diet HY + RU or control diet HY—RU. (F) Average median lifespan from multiple repeats of the fly strains shown in A-D fed the different HY diets. Error bars represent the SE. (TIF) [file pgen.1008318.s005.tif]
