# Supplementary material for: A conserved role of the insulin-like signaling pathway in diet-dependent uric acid pathologies in Drosophila melanogaster
Source: PLoS Genet. 2019 Aug 15;15(8):e1008318. doi: 10.1371/journal.pgen.1008318 (PMC6695094; doi:10.1371/journal.pgen.1008318)
Supplement: S1 Table — Serum uric acid (SUA) levels are not included in a typical doctors’ visit and are therefore missing for most healthy controls. Avg, average; max BMI, maximal body mass index in the evaluation period. (DOCX) [file pgen.1008318.s006.docx]

**Tab S1**

|  | Phenotype | | |
| --- | --- | --- | --- |
|  | Gout | | Avg  SUA |
|  | Cases | Controls |  |
|  | Number (Percent) | | |
| Male | 3426 (74.3) | 30400 (40.5) | 13567 (52.0) |
| Female | 1182 (25.7) | 44581 (59.5) | 12536 (48.0) |
| Gout | 4608 (100) | 0 (0) | 4015 (15.4) |
| Diuretics | 3390 (73.6) | 29769 (39.7) | 14846 (56.9) |
|  | Mean ± SD | | |
| Average SUA | 8.10 ± 1.6 | 5.62 ± 1.5 | 6.03 ± 1.8 |
| Max BMI | 31.25 ± 6.0 | 28.51 ± 5.8 | 29.91 ± 6.2 |
