# Supplementary material for: A conserved role of the insulin-like signaling pathway in diet-dependent uric acid pathologies in Drosophila melanogaster
Source: PLoS Genet. 2019 Aug 15;15(8):e1008318. doi: 10.1371/journal.pgen.1008318 (PMC6695094; doi:10.1371/journal.pgen.1008318)
Supplement: S2 Table — SNPs reaching the gene-specific significance threshold are shown with their effect sizes and specific P-values. SNP, single nucleotide polymorphism; SE, standard error; OR, odds ratio; UA, uric acid. (DOCX) [file pgen.1008318.s007.docx]

**Tab S2**

| SNP | Effect | SE | OR/BETA | P-value | Gene | Phenotype |
| --- | --- | --- | --- | --- | --- | --- |
| rs187303420 | 0.5881 | 0.1268 | OR | 2.82E-05 | *AKT2* | Gout |
| rs72940696 | -0.1787 | 0.0435 | BETA | 4.04E-05 | *FOXO3* | Avg UA |
| rs3800232 | -0.0746 | 0.0217 | BETA | 5.89E-04 | *FOXO3* | Avg UA |
