# Supplementary material for: A conserved role of the insulin-like signaling pathway in diet-dependent uric acid pathologies in Drosophila melanogaster
Source: PLoS Genet. 2019 Aug 15;15(8):e1008318. doi: 10.1371/journal.pgen.1008318 (PMC6695094; doi:10.1371/journal.pgen.1008318)
Supplement: S3 Table — (DOCX) [file pgen.1008318.s008.docx]

| **Analyte** | **Ionization mode** | **Precursor ion (Q1)** | **Product ion (Q3)** | **Retention time (min)** | **Declustering potential (V)** | **Entrance potential (V)** | **Collision energy (V)** | **Collision exit potential (V)** |
| --- | --- | --- | --- | --- | --- | --- | --- | --- |
| Adenosine | Positive | 268 | 119 | 1.6 | 40 | 10 | 57.4 | 10 |
|  | Positive | 268 | 94 | 1.6 | 40 | 10 | 49.9 | 6.5 |
| Adenine | Positive | 136 | 119.2 | 2 | 40 | 10 | 29.8 | 10 |
|  | Positive | 136 | 94 | 2 | 40 | 10 | 36.6 | 10 |
| Allantoin | Negative | 157.1 | 114 | 2.2 | -40 | -10 | -18.9 | -8 |
|  | Negative | 157.1 | 97 | 2.2 | -40 | -10 | -19.7 | -8 |
| Guanine | Positive | 152 | 135 | 2.9 | 40 | 10 | 24 | 10 |
|  | Positive | 152 | 93 | 2.9 | 40 | 10 | 27 | 10 |
| Hypoxanthine | Positive | 137 | 119 | 3.7 | 40 | 10 | 26.5 | 10 |
|  | Positive | 137 | 110 | 3.7 | 40 | 10 | 26.8 | 10 |
| Guanosine | Positive | 284 | 152 | 4.1 | 40 | 10 | 20.5 | 10 |
|  | Positive | 284 | 135 | 4.1 | 40 | 10 | 48.4 | 10 |
| Inosine | Positive | 269 | 137 | 4.6 | 40 | 10 | 16 | 10 |
|  | Positive | 269 | 133 | 4.6 | 40 | 10 | 15 | 10 |
| Xanthine | Negative | 151 | 108 | 6.7 | -40 | -10 | -23 | -8 |
|  | Negative | 151 | 42 | 6.7 | -40 | -10 | -39 | -8 |
| Urica acid | Negative | 167 | 124 | 8 | -40 | -10 | -20 | -10 |
|  | Negative | 167 | 96 | 8 | -40 | -10 | -24.5 | -10 |
| Adenosine monophosphate | Negative | 346.1 | 79 | 8.6 | -40 | -10 | -72 | -10 |
|  | Positive | 348 | 136 | 8.6 | 40 | 10 | 23 | 10 |
| Guanosine monophosphate | Positive | 364 | 152 | 9.2 | 40 | 10 | 17 | 11 |
|  | Negative | 362 | 79 | 9.2 | -40 | -10 | -77 | -10 |
| Inosine monophosphate | Negative | 347 | 211 | 9.5 | -40 | -10 | -23 | -13 |
|  | Positive | 349 | 137 | 9.5 | 40 | 10 | 21 | 10 |
| 2-Chloro-adenosine (Internal Standard) | Positive | 302 | 169.9 | 1.3 | 40 | 10 | 22 | 10 |
|  | Positive | 302 | 134 | 1.3 | 40 | 10 | 52 | 10 |

**Tab. S3**
